# *Viburnum opulus* L.—A Review of Phytochemistry and Biological Effects

**DOI:** 10.3390/nu12113398

**Published:** 2020-11-05

**Authors:** Dominika Kajszczak, Małgorzata Zakłos-Szyda, Anna Podsędek

**Affiliations:** Institute of Molecular and Industrial Biotechnology, Faculty of Biotechnology and Food Sciences, Lodz University of Technology, 90-924 Łódź, Poland; anna.podsedek@p.lodz.pl

**Keywords:** *Viburnum opulus* L., nutrients, secondary metabolites, biological activity

## Abstract

*Viburnum opulus* (VO) is a valuable decorative, medicinal, and food plant. This deciduous shrub is found in natural habitats in Europe, Russia, and some regions in North Africa and North Asia. The VO is traditionally used to treat aliments such as cough, colds, tuberculosis, rheumatic aches, ulcers, stomach, and kidney problems, among others. Many of the health-promoting properties of VO are associated with antioxidant activity, which has been demonstrated in both in vitro and in vivo studies. The results of in vitro studies show the antimicrobial potential of VO, especially against Gram-positive bacteria. In cell-based studies, VO demonstrated anti-inflammatory, anti-obesity, anti-diabetic, osteogenic, cardio-protective, and cytoprotective properties. The applicability of VO in the treatment of urinary tract diseases, endometriosis, and some cancers has been confirmed in in vivo studies. The health benefits of VO result from the presence of bioactive components such as phenolic compounds, vitamin C, carotenoids, iridoids, and essential oils. The aim of this review is to present an overview of the botanical characteristics, chemical compositions, including bioactive compounds, and pro-health properties of VO different morphological parts.

## 1. Introduction

*Viburnum opulus* (VO) is a plant that belongs to the *Viburnum* L. genus from Adoxaceae family, sometimes included in the monotypic family Viburnaceae, formerly also for the Caprifoliaceae. It is known as guelder rose, European guelder, European cranberrybush, water elder, rose elder, Rose Ebru, cherry-wood, crampbark, snowball tree, and as gilaburu in Turkey [1,2,3,4,5]. VO is common in natural habitats on the European continent and in some regions of North Africa and North Asia, and also in the central zone of Russia [6,7,8].

VO is a valuable decorative, medicinal, and food plant. In Russia and Ukraine nations the red VO fruits, despite their astringent-bitter-sour taste, are used in traditional cuisine as a component of e.g., marmalades, jams, cordials and liqueurs, and “Kalinnikov” pies as well as herbal teas [9]. Also, in Scandinavia the fruits are popular when cooked into preserves while in Canada they may replace cranberries [4]. In the Kayseri region of Turkey, VO fruits are allowed to stand in plastic drums containing tap water at a dark place and room temperature approximately for 3–4 months to ferment and eliminate the sour, pungent flavor [10,11]. The gilaburu juice is a traditional non-alcoholic fermented beverage and is available in the commercial offer. Recently, research has been carried out on the development of VO for the production of functional food such as a pear juice with the addition of VO juice, or cakes incorporated with VO fruit pomace [12,13]. The results of the studies by Çemtekin et al. [14] suggested the possibility of using VO fruit concentrate in cooked minced turkey as an alternative antioxidant source for nitrites and butylated hydroxytoluene to delay oxidative changes. 

The VO is widely used for medicinal purposes. The gilaburu juice is traditionally used to treat ailments such as cough, colds, tuberculosis, rheumatic aches, ulcers, liver disease, diabetes, and hypertension as well as to prevent some stomach and kidney problems [6,11,13,15,16]. The bark of VO (*Cortex Viburni*) is used in the treatment of stomach or uterine bleeding and hemorrhoids [17]. 

The results of published in vitro studies indicate antimicrobial [18,19], antidiabetic [20,21,22], anti-obesity [23,24], anti-inflammatory [25,26], and anti-cancer [27,28,29] properties of different morphological parts of VO. In animal studies, a beneficial effect on the urinary system [30,31], anti-inflammatory [6], and vasorelaxant [32] activities of VO was demonstrated. 

Health benefits of VO result from the presence of bioactive components such as phenolic compounds, vitamin C, carotenoids, iridoids, and essential oils, among others [8,9,33,34,35,36]. This review article summarizes current knowledge and the available information of *Viburnum opulus* for its botanical characteristics, chemical compositions, including bioactive compounds, and pro-health properties demonstrated by in vitro and in vivo assays. 

## 2. Botanical Characteristics 

VO is the most popular variety in Europe, with the exception of the northern ends of the Scandinavian Peninsula and the southern ends of the Iberian, Apennine, and Balkan Peninsulas [9,17]. In addition, two others varieties such as *V. opulus* var. *americanum* Aiton growing in North America and *V. opulus* var. *sargentii* (Koehne) Takeda native to Korea, Northern China, and Japan have been recognized as the same species [33].

**Figure 1:**
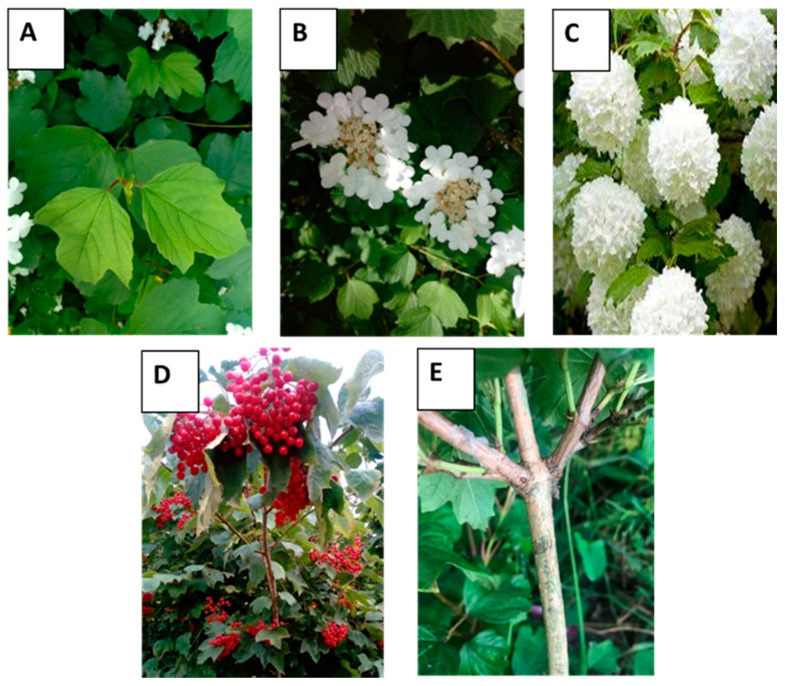
Leaves (**A**), flowers of *edule* variety (**B**), and flowers of decorative variety (**C**), fruits (**D**), and bark (**E**) of *V. opulus* growing in central Poland.

VO is a fast-growing, up to 4–5 m height, deciduous shrub. Its leaves are opposite, three-lobed, with a rounded base and coarsely serrated margins (Figure 1A). On the top, they are naked and dark green, while underneath they are lighter, slightly hairy with star-shaped hairs. The leaves develop together with the flowers and then turn discolored into scarlet-purple. 

The white flowers are produced in corymbs 4–11 cm in diameter at the top of the stems. Each bloom is composed of an outer ring of large sterile flowers (1.5–2.5 cm in diameter) and an inner ring of tiny fertile ones (4–5 mm) (Figure 1B). The flowers bloom in late spring, and are pollinated by insects [17]. The decorative cultivar “Roseum” (synonym “Sterile”, “Snowball”) has only sterile type of flowers that give the appearance of snowballs (Figure 1C).

The shiny and spherical VO fruits have light red, red, or dark red skin color (Figure 1D). They can be harvested from late summer to mid-autumn, and by winter the fruits shrivel and look like dried red raisins. The fruits are bitter with a strong astringent taste, and they emit a strong unpleasant odor [2,7,37,38,39]. The productivity of fruits depends on the cultivar and varies from 29.0 to 79.0 of fruits per raceme. The weighting of one fruit is within the range from 0.40 to 1.80 g. The length of the fruits are 1.04–11.85 mm and their width vary from 1.02 to 9.60 mm [2,5,7,33,39,40,41,42,43,44]. In the yellow flesh of fruits, there is one oval or heart-shaped seed with about 30.5–112 mg weight [2,7,39,42]. The bark of VO is green-brown on the outer surface and green-yellow to red-brown on the inner surface (Figure 1E). The bark is harvested in spring and summer when the plant is flowering. Like fruit, it has a strong characteristic odor and tastes somewhat bitter.

## 3. Macronutrients, Minerals, and Dietary Fiber Composition

So far, most of the research has been carried out to characterize the chemical composition of VO fruits [2,5,8,9,18,33,34,36,39,40,42,43,44,45]. However, few reports concern the composition of other morphological parts of the plant, such as the bark, stalks, flowers, and leaves [34,35,46,47,48]. The main component of VO fruits is water, which makes up to 85.7–88.3% of their fresh weight [5,33]. The total soluble solids (TSS) content varied from 9.0 to 24.7% [33,40,42,43,45]. The sugars are one of the main groups of fruit substances to be synthesized in the plant from simple organic compounds. The reducing sugar content of VO fresh fruit ranged between 4.02 and 8.80% while sucrose content varied from 0.13 to 140 mg/100 g, and the ratio of glucose content to the fructose content was from 1.0 to 1.5 [2,5,9,33]. For comparison, the total sugars of VO-dried flowers and bark were near three times and above twenty times lower than in dried fruits. In addition, bark contained only sucrose and this disaccharide dominated in flowers (47.4% of total sugars) [34].

The fruits of VO are highly acidic because of the large content of various acids. Their titratable acidity was between 1.34 and 3.20% [5,33,45]. The content of organic acids of Turkish VO fruits was 0.25–1.25% of fresh weight [2,40,41]; and from 0.65 to 3.86% for the fruits harvested in Russia [9]. The organic acids profiles of VO fruits are shown in Table 1. Malic and citric acids were the main organic acids present in the fruits from Russia while malic and tartaric acids dominated in fruits from Turkey. The content of organic acids in VO bark and flowers was 1.84 and 1.81 g/100 g dried weight, respectively with succinic acid (52.7% of total content) and malic acid (33.7% of total content) as the dominant compound [34]. Malic, citric, and quinic acids were also reported in VO fruit pomace [49]. Cam et al. [50] determined only oxalic acid and malic acid in VO seeds in the amount of 0.56 g and 0.26 g per 100 g, respectively. The content of other nutrients (protein and lipids) depends on the parts of the VO plant. The flowers (9.72 g/100 g dried weight) were three or two times abundant in protein compared to the bark or fruits, respectively [34]. Protein results of VO fruits, stalks, and leaves were determined as 5.67–6.71 and 12.10%, respectively [5,44,47]. While the protein content in VO seeds was 5–6% with glutamic and aspartic acids, as well as arginine and leucine as the main amino acid [51]. For comparison, the content of protein in VO fruit pomace was 16.82% [49]. The levels of lipids were comparable in VO-dried fruits and bark (about 10 g/100 g), while the dried flowers contained about two times less. Similar lipid contents in VO fruits (9.34 to 12.35% of dry weight) were given by Zarifikhosroshahi et al. with oleic acid, linoleic acid, and palmitic acid as the major fatty acids [36]. In addition, unsaturated fatty acids were shown to predominate over saturated fatty acids in VO fruits. Higher total lipid content (10.3–13.3%) was marked in 14 VO genotypes collected from the Gumushane province of Turkey [44]. The major class of compounds present in nonpolar lipid fraction was triacylglycerides (93.1% of the whole mass of nonpolar lipids) [51]. This fraction also contained esters of triterpenes, mono- and diacylglicerydes, sterols, free fatty acids, and triterpene alcohols. Polar lipid fraction consisted of glyco- and phospholipids the concentration of which was estimated at 91.8 and 12.2 mg/100 g dry weight of fruits, respectively. Additionally, the authors showed that the lipids determined in the fruits are present mainly in the seeds because the pulp contained only nonpolar lipids which accounted for only 0.04% of its weight. For comparison, the oil content in VO seeds was 12.1% and VO fruit pomace contained 26.24% fat [49]. Yilmaz et al. [52] identified 21 fatty acids in VO seeds with oleic and linoleic acids as the major fatty acids. In VO leaves following triterpenes were identified: α- and β-amyrin, along with sterols: campesterol, stigmasterol, and β-sitosterol while in VO flowers only β-sitosterol and ursolic acid [35]. 

VO fruits own some typical smell notes, which unfortunately are rather disliked by the consumers. The volatile constituents identified in the fruits belong to the following classes of organic compounds: alcohols, terpenoids, phenols, ketones, aldehydes, esters, branched fatty acids, and acids [36,53]. The researchers found 23–44 volatile compounds depending on the assay used. 3-Methylbutanoic acid, 2-methylbutanoic acid, as well as linalool and ethyl decanoate were found to be the main odor active components of VO fruits aroma. The odor of these compounds was described as possessing strong “old cheese” and “dirty socks” [53]. Sönmezdaǧ et al. [54] and Yilmaztekin and Sislioglu [55] examined volatile compounds of fermented VO fruits known as glilaburu juice. They identified up to 58 compounds and reported that acid compounds such as isovaleric acid, butanoic acid, and propanoic acid were the dominant volatile compounds in non-alcoholic fermented juice. 

The content of minerals in different morphological parts of VO varied [47]. With respect to macroelements VO fruits, leaves, and stalks contain potassium, calcium, phosphorus, magnesium, and sodium. From the group of microelements, the content of iron, copper, zinc, and manganese was determined. The results showed that the minerals contents of the leaves (except cooper) were higher than the fruits and stalks. VO fruits are notable for their potassium content (842–930 mg/100 g of fresh weight) [5,47]. Potassium intake helps to maintain cardiovascular health and muscle function by regulating the blood pressure through the modulation of liquid retention in the body [56]. 

As with nutrients, also the content and composition of soluble and insoluble dietary fiber fractions varied with parts of VO plant [34]. The total dietary fiber content per 100 g of dry weight was 38.44 g in fruits, 45.39 g in flowers, and 59.34 g in bark. The insoluble dietary fiber constitutes 82.3%, 93.5%, and 98.1% of the total amount of fiber in fruits, flowers, and bark, respectively. In all analyzed parts of VO plant, the insoluble dietary fiber was dominated by Klason lignins while the soluble dietary fiber in fruits and bark by neutral sugars fraction, and by the uronic acid fraction in flowers. Pectins are part of the soluble dietary fiber and their total amount varied significantly from 4.15 to 8.58 g/100 g of VO bark and flowers dry weight, respectively. Flowers and bark were dominated by ionically cross-linked pectin as reflected by the chelator-soluble pectin value while pectin of VO fruits mainly consisted of water-soluble pectin.

## 4. Antioxidative Components 

Plants are known as a natural source of different compounds with antioxidant properties such as ascorbic acid (vitamin C), α-tocopherol (vitamin E), carotenoids, chlorophylls, and phenolic compounds [57]. Antioxidants may protect human cells from reactive oxygen species (ROS) and reactive nitrogen species (RNS) generation of which is exacerbated under pathological conditions in different ways. Antioxidants can convert free radicals into non-radical compounds, break the chain reaction of lipid oxidation, inhibit pro-oxidative enzymes and chelate metal ions, among others. So, antioxidants present in the diet may have a significant effect on the prophylaxis and progression of various diseases associated with oxidative stress.

Water-soluble vitamin C is present in the cellular fluids such as cytosol, or cytoplasmic matrix but the lipid-soluble vitamin E and carotenoids are predominantly located in cell membranes [58]. Vitamin C can act directly by reaction with aqueous peroxyl radicals, and indirectly by restoring the antioxidant properties of fat-soluble vitamin E. Vitamin E and carotenoids function as a “chain breaker” during lipid peroxidation in cell membranes and various lipid particles including low-density lipoprotein. Vitamin C content in the VO fruit was varied and ranged from 12.4 to 164 mg/100 g of fresh weight depending on the place of cultivation and genotypes. The Turkish genotypes of VO fruits exhibited vitamin C content between 25.0 and 59.5 mg per 100 g fresh fruits [2,5,40,43,50,59]. Česonienė et al. [33] and Perova et al. [9] reported greater variation in vitamin C content (12.4–92 mg/100 g) in Lithuania genotypes. However, fruits produced in Ukraine contained from 43.1 to 75.2 mg of vitamin C per 100 g [45]. The fruits harvested in the territory of the Czech Republic turned out to be the most abundant in this vitamin (101–164 mg/100 g) [8]. The content of total carotenoids expressed as β-carotene equivalents in VO fruits varied from 1.4 to 2.8 mg/100 g fresh weight [7,33]. On the other hand, the content of beta-carotene determined by paper chromatography followed by photoelectrocolorimetry in the fruits of six VO varieties ranged from 0.21–0.51 mg/100 g [45]. Our previous study showed that dried VO fruits contained above twice more total carotenoids (2.70 mg/100 g dry weight) than dried VO bark and flowers [34]. To our best knowledge, there are no data available on the vitamin E content of VO fruit. It was only reported, that the oil isolated from VO seeds originated from Siberia was rich in α-tocopherol which was accompanied by γ-tocopherol and δ-tocopherol (110, 40, and 60 mg/100 g oil, respectively) [60].

Phenolic compounds represent a large group of secondary plant metabolites that possess one or more aromatic rings bearing one or more hydroxyl groups. A huge variety of phenolics are produced by plants, with 1000s recognized throughout the plant kingdom. Their structures may range from that of a simple phenolic molecule (i.e., phenolic acids and phenolic alcohols) to that of a polyphenol structure (i.e., stilbenes, flavonoids) and a complex high-molecular mass polymer (hydrolyzable and condensed tannins). Additionally, they occur in free and conjugated forms with acids, sugars, or other water-soluble or fat-soluble compounds [61]. Current literature suggests that the long-term consumption of diets rich in polyphenols protects against certain cancers, cardiovascular diseases, type 2 diabetes, osteoporosis, pancreatitis, gastrointestinal problems, lung damage, and neurodegenerative diseases [62].

There are several research studies about phenolics content and composition of VO fruit but very few literature data concerning the composition of the phenolic compounds of VO bark, leaf, and flower. The total content of different sub-groups of phenolic compounds in VO is presented in Table 2.

The data in Table 2 showed that the content of total phenolics in VO fruit was in the range of 325.4–1460 g/100 g of fresh weight. Their concentration in VO seed exceeded the concentration in the most fruit genotypes. According to Polka et al., VO bark was characterized by a higher level of total phenolics as compared to fruit and flower [34]. Total flavonoids in VO fruits were between 187 and 489 g per 100 g fresh weight and they constituted from 21.73% to 57.16% of the total content of phenolic compounds. In seeds, bark, and flowers of VO, flavonoids accounted for 83.86%, 56.53%, and 47.58% of total phenolics, respectively. The VO fruits had a red skin color because of the presence of water-soluble anthocyanins and lipid-soluble carotenoids. The amounts of total anthocyanins in VO fruits expressed as cyanidin 3-rutinoside ranged from 6 to 53 mg/100 g fresh weight and expressed as cyanidin 3-glucoside from 23 to 45 mg/100 g fresh weight. They accounted from 0.82 to 20.11% of total phenolics and for most of the analyzed genotypes, it was within the range of 2.03–7.04%. According to Perova et al. [9], proanthocyanidins are a quantitatively significant component of the fresh VO fruits with content 201–528 mg/100 g fresh weight and they accounted for 49.9–2.8% of total phenolics. In our previous study, total proanthocyanidins in VO-dried fruits, bark, and flowers accounted only for 13.9%, 25.9%, and 6.3% of total phenolics, respectively [34].

The source of phenolic compounds in our diet may also be the fermented beverage and juice obtained from VO fruits. A non-alcoholic beverage is a traditional drink for people living in Kayseri city and the middle Anatolian region of Turkey [5]. For the preparation of the beverage, the fruits are left in water in a dark place at room temperature for about 3–5 months to ferment and produce a non-alcoholic fermented product [3,10]. The content of total phenolics in the juice after 4 months of fermentation was 483 mg of gallic acid equivalents/100 g of juice and was higher by 16.9% than in the raw juice before fermentation [55]. For comparison, fresh juice obtained from shredded and preheating to 90 °C VO fruits followed by straining through a pulper finisher contained 590.3 mg of total phenolics/100 mL [4]. Higher amounts of phenolic compounds were found in the juice pressed from ground fresh whole fruits in a hydraulic press (3823 mg/100 mL) and in the juices pressed from the lightly crushed fresh fruits without stone seeds using a hand press (880.1–1168.8 mg/100 g) [33]. However, the juices obtained from crushed fresh fruit by centrifuging the mash (5000 rpm for 10 min) contained 598 mg of total phenolics per 100 g [21], while produced from crushed defrosted fruit by centrifuging the mash (10,000 rpm for 15 min) contained 547 to 630 mg of total phenolics per 100 g, depending on the fruit genotype [38].

Apart from determining the general content of phenolic compounds, it is very important to determine their qualitative composition because of their huge structural diversity that significantly affects their properties. Generally, VO has a diverse phytochemical profile with phenolic acids such as hydroxybenzoic and hydroxycinnamic acids, three classes of flavonoids (i.e., flavonols, flavanols, and anthocyanins). The overview of phenolic compounds identified in VO fruits is shown in Table 3 and in VO fruit juice in Table 4. The results showed significant differences in phenolic composition between the studied VO fruits genotypes harvested in Lithuania [9] and in Turkey [2,4]. So far, three hydroxybenzoic acids, five hydroxycinnamic acids, three flavanols, nine flavonols, and ten anthocyanins were identified in VO fruits—Table 3. In the above-mentioned groups, quantitatively dominant compounds are gallic acid, chlorogenic acid, quercetin 3-sambubioside, and cyanidin 3-xylosyl-rutinoside with cyanidin 3-glucoside, respectively. However, the results of quantitative analysis are controversial. According to Velioglu et al. [4] and Perova et al. [9] chlorogenic acid was the main component of VO fruits. Additionally, the analysis of the phenolic composition of VO fruit pomace showed the presence of other phenolic acids (dihydroferulic acid 4-glucuronide, dihydroksybenzoic acid derivative, and ethylchlorogenate), flavanols ((epi)catechin dimer monoglycoside and (epi)catechin-dihexoside), as well as five flavalignans (cinchonain derivatives), and other new flavonoids (scopoletin-7-O-sophoroside, dihydromyricetin, and gambiriin) [49].

On the other hand, Özrenk et al. [2] indicated (+)-catechin and gallic acid as the most abundant components in VO fruits while chlorogenic acid concentration was 8–10 times lower than the (+)-catechin content. As with fruits, chlorogenic acid showed the highest content in juice followed by procyanidin B1 and (+)-catechin. VO fruits are characterized by varied content of anthocyanins, cyanidin 3-xylosyl-rutinoside, cyanidin 3-glucoside, and cyanidin 3-arabinosyl-glucoside as the main pigments among ten identified compounds (Table 3). Zakłos-Szyda et al. [24] identified only three anthocyanins in VO juice with cyanidin 3-glucoside as the dominant pigment (Table 4). Among the identified and estimated phenolic compounds, flavonols occurred at the lowest concentration in both VO fruit and juice. 

Compared to VO fruit, studies on the phenolic profile of other morphological parts of VO are very sparse. The previous research using the ultra-performance LC system has shown the presence of hydroxycinnamic acids (chlorogenic, neochlorogenic, cryptochlorogenic), flavanols (catechin, procyanidin B1), and flavonols (quercetin 3-rutinoside and glucoside, isorhamnetin, isorhamnetin 3-glucoside) in VO flowers, while only flavanols (catechin, epicatechin, procyanidin B1 and B2) and hydroxycinnamic acids (chlorogenic, neochlorogenic, cryptochlorogenic p-coumaric) in VO bark [34].

Additionally, the results showed that hydroxycinnamic acids dominated in the VO flowers and flavanols in VO bark. Turek and Cisowski [48] using high-pressure liquid chromatography (HPLC) as well as electrospray ionization time-of-flight mass spectrometry (ESI-TOF MS) and proton nuclear magnetic resonance (^1^H-NMR) methods have reported the presence of chlorogenic acid as the free acid and many acids liberated from esters and glycosides after alkaline and enzymatic hydrolyses such as gallic, 4-hydroxybenzoic, protocatechuic, 3,4-dixydroxyphenylacetic, 3,4,5-trimetoxybenzoic, syringic, homogentisic, caffeic, ferulic, *p*-coumaric, and ellagic acids. Altun and Yilmaz [63] determined salicin and chlorogenic acid in VO leaves and branches. Dried leaves contained 0.90% of salicin and 0.68% of chlorogenic acid. By comparison, dried branches had more salicin (1.25%) but less chlorogenic acid (0.36%). 

VO also contains iridoids—organic molecules from the monoterpenoid group. They are found in the green parts of plants, mainly in the leaves and young stems, and sometimes in fruits and sprouts. Iridoids show pro-health effects, including anticancer, cardiovascular, hypolipidemic, antiviral, and immunomodulatory activities. The health-improving properties of iridoids are majorly due to their antioxidative and anti-inflammatory properties [64,65,66].

To our best knowledge, the structure of the VO iridoids has been described in only three publications [9,49,67]. The first one concerns opulosides I-IV isolated from VO leaves, the second one opulosides I-IV, and additionally opuloside X present in VO fruits. The latest study on VO fruit pomace revealed the presence of six iridoids including secologanate and viburtioside derivatives [49]. Opulosides are rare types of iridoid structures whose carbohydrate part consists of allose and xylose instead of glucose, as is the case with most iridoids.

## 5. *Viburnum opulus* Health-Promoting Effects

### 5.1. Antioxidative Effect

Reactive oxygen species (ROS) produced intracellularly act as subcellular messengers in signal transduction pathways or as a part of the cell defense mechanism [68]. However, their excessive production can result in damage of many molecules, including protein and lipid peroxidation, DNA strand breaks, and RNA modifications, which further generate intracellular oxidative stress. The alteration of intracellular homeostasis manifests with cell dysfunction, metabolic failure, and finally, cellular death induction. The intake of exogenous antioxidants, such as vitamins C and E, phenolic compounds, and natural plant pigments, may support the antioxidative defense [69]. 

To evaluate the antioxidant ability of single compounds and complex extracts from plants, foods, and biological samples, several in vitro assays, including chemical and cellular methods have been frequently used [70,71,72]. Considering the mechanism underlying the antioxidant-oxidant reaction, the chemical methods were divided into hydrogen atom transfer (HAT, i.e., ABTS, DPPH, ORAC, and TRAP methods) and single electron transfer (SET, i.e., DPPH, ABTS, FRAP, and CUPRAC methods) techniques. HAT-based methods measure the capacity of an antioxidant to trap free radicals by hydrogen donation, while SET methods rely on the one electron transfer reductive ability of an antioxidant compound versus a radical species. In systems containing a lipid substrate, the effect of antioxidants on the formation of oxidation products (i.e., hydroperoxide, conjugated diene, or thiobarbituric acid reactive substances) is determined. In addition to the above-mentioned methods, various cellular models are also used to assess the antioxidant activity and the basic mechanisms of oxidative stress, as well as to explain the mechanisms of action of antioxidants on various oxidative stressors. In various culture systems, antioxidants are added to the cell culture medium simultaneously with the stressor or the cells are pre-incubated with the antioxidant to allow its incorporation into the cells. In this method, intracellular redox status is repaired by scavenging free radicals with the addition of antioxidant substances. Consequently, with the application of this method, changes in physiological conditions can be simulated by the changes of cell morphology, cell survival rate, ROS level, antioxidant enzymes, and lipid peroxides.

A number of in vitro studies describe the antioxidant potential of VO fruits (Table 5), while other parts of the plant, such as the bark, leaf, branch, and flower, have been assessed to a lesser extent. The antioxidant potential of the various morphological parts of VO and the extracts obtained from them was determined by various methods. The authors of the published studies used various extraction solvents, such as water, acetone, methanol, and ethanol, as well as aqueous solutions of organic solvents without or with the addition of inorganic or organic acid, to separate antioxidants from the tested plant material. In addition, the antioxidant capacity was determined as the half maximal inhibitory concentration (IC_50_ or EC_50_), either in Trolox, ascorbic acid, or butylated hydroxytoluene equivalents (e.g., in the DPPH method—Table 5). According to Barak et al. [73], the extraction with 96% methanol was more effective than that with water in antioxidants extractions from VO fruit. The methanol extract was more potent scavengers of DPPH^●^ radical and showed higher potential for reducing iron(III) and copper(II) ions, and was also characterized by a higher total antioxidant capacity than water extract. On the other hand, the water extract exceeded the methanolic extract by 5% in the scavenging efficiency of *N*,*N*-dimethyl-ρ-phenylenediamine (DMPD). In turn, the decreasing rank of antioxidant capacity of fruit pomace extracts due to the type of extractant is as follows: water> acetone> ethanol regardless of the type of antioxidant potential determination method (DPPH, ABTS, and ORAC) [74]. The ferrous ion chelating capacity of VO fruit extracts was the highest when ethyl acetate was used as the extraction solvent and the lowest for an aqueous extract [75]. Considering the antioxidant potential of different parts of the VO, the bark extractable components exceeded the fruit and flower in terms of scavenging free radicals (ABTS^•+^ radical cation, ^•^OH hydroxyl radical, O_2_^•−^ superoxide anion radical and peroxyl radical), and reducing ferric to ferrous ion [76]. The aqueous extract of VO leaf was more efficient scavengers of DPPH^●^ and O_2_^•−^ superoxide anion radicals than the aqueous extract of the branch [6]. The same relationship was found for the ability of VO leaf and branch acetate extracts to chelate ferrous ion, and the opposite when methanol and water was used as the extraction solvents [75].

The antioxidant properties of VO have also been demonstrated in various cell models such as human adenocarcinoma Caco-2, human hepatoma HepG2, human neuronal cells SH-SY5Y, mouse pancreatic cells MIN-6, and mouse preadipocytes 3T3-L1 [20,23,24,77]. Zakłos-Szyda et al. [21] reported that VO fruit fresh juice and juice stripped of non-phenolic polar compounds removed on the Sep-Pak C18, were able to decrease intracellular ROS level by 20% at 50–75 µg/mL dose during in vitro studies in human adenocarcinoma Caco-2 cells. These antioxidative properties are especially relevant for metabolically active cells, like secreting insulin or undergoing differentiation because during these processes generation of large quantities of radicals occurs [78,79]. In other studies, it was observed that VO fruit juice enriched with phenolics (50–75 µg/mL) decreased intracellular ROS level in mouse insulin-secreting pancreatic MIN-6 cells by 25%, as well as in mouse differentiated adipocytes 3T3-L1 [22,24]. Comparable ability was observed in 3T3-L1 cells for VO-dried fruit acetone extract and semi-purified phenolic extract which at concentration 75 µg/mL reduced intracellular ROS production by 20% [23].

Under in vitro conditions, intracellular oxidative stress can be chemically induced at defined conditions by *tert*-bytulhydroperoxide (*t*-BOOH) or H_2_O_2_*. t*-BOOH can be metabolized via cytochrome P-450 leading to the production of ROS attacking cellular proteins or membrane phospholipids, whereas H_2_O_2_ is converted, via the Fenton reaction, to the most toxic ^•^OH radicals in the presence of ferrous ions [80,81]. Fresh VO fruit juice, as well as juice enriched with phenolics at concentration 50–75 µg/mL, showed protective activity against oxidative stress generated by *t*-BOOH in human hepatoma Hep-G2, insulin-secreting β-TC3 and MIN-6 cells, as well as partially restored their metabolic activity [20,22]. Paşayeva et al. demonstrated the protective effect of ethanol extract, decoction, and juice from VO fruits on H_2_O_2_ induced oxidative stress in human neuronal SH-SY5Y cells, however the more detailed data according to the molecular mechanism of action were not present [77].

The antioxidant properties of VO were also demonstrated with established animal models. Zayachkivska et al. investigated the influence of proanthocyanidins isolated from air-dried VO fruits on gastrointestinal mucosal damage induced by water immersion and restraint stress in male Wistar rats [83]. The results showed that VO proanthocyanidins applied orally in doses of 25, 50, or 75 mg/kg body weight increased the activity of superoxide dismutase (SOD), catalase (CAT), and glutathione peroxidase (GPx) in gastric mucosa, inhibited the production of ROS, and decreased the expression by tissue concentration of malondialdehyde comes from lipid peroxidation. These enzymes involve in ROS breakdown to harmless molecules [80]. SOD is the most active enzyme which catalyzes the dismutation of two molecules of superoxide anion to hydrogen peroxide and molecular oxygen. CAT completes the detoxification process initiated by SOD via degradation and reduction of hydrogen peroxide to water and molecular oxygen. GPx converts hydrogen peroxide into water or lipid peroxides to their corresponding alcohols, and therefore plays a crucial role in the inhibition of the lipid peroxidation process. Eken and co-workers investigated the effect of VO-dried fruit components against ischemia-reperfusion-induced oxidative stress during the lung transplantation in female Wistar rats [84]. In the group with induced oxidative stress the SOD, CAT, GPx activities, and total glutathione levels were markedly lower, whereas VO treatment compensated this effect. Malondialdehyde level in the lung tissue, which corresponds to lipid peroxidation of cellular membranes, as well as level of plasma protein carbonyl, were reduced in rats treated with VO fruit extract. These findings indicated that VO can be used as a therapeutic against oxidative stress during lung transplantation. 

### 5.2. Antimicrobial Activity 

Because of the increase of bacteria strains with multi-resistance against antibiotics, there is a need of finding of plant-originated agents with antimicrobial activity. These phytocompounds can be used as the components of medicinal treatments, as well as to preserve the growth of microorganisms in food products. So far, VO has been demonstrated to possess antibacterial effects against several pathogenic Gram-positive and Gram-negative bacteria. Methanol extract obtained from seedless VO-dried fruits at 10% concentration effectively inhibited growth of *Aeromonas hydrophila* ATCC7965, *Bacillus cereus* FMC 19, *Enterobacter aerogenes* CCM 2531, *Escherichia coli* DM, *Klebsiella pneumoniae* FMC 5, *Proteus vulgaris* FMC 1, *Pseudomonas aeruginosa* ATCC 27853, *Salmonella typhimurium*, *Staphylococcus aureus* Cowan 1, and *Yersinia enterocolitica* EU [18]. For comparison, the 2% concentration of extract did not inhibit the growth of any bacteria. It was demonstrated that the most sensitive was *Aeromonas hydrophila*, whereas the most resistant bacterium was *Yersinia enterocolitica.* Other studies have shown that the ethanol extracts and juices obtained from fruits of five VO genotypes displayed antibacterial activity toward *Pseudomonas aeruginosa*, *Escherichia coli*, *Salmonella typhimurium*, *Salmonella agona*, *Staphylococcus aureus*, *Bacillus subtilis*, *Listeria monocytogenes*, *Enterobacter faecalis*, *Micrococcus luteus*, *Staphylococcus epidermidis,* as tested by the disc diffusion method [15]. The most effective antibacterial activity was exhibited by the fruit juices against Gram-positive bacteria such as *Salmonella typhimurium*, *Salmonella agona*, and *Listeria monocytogenes*. Moreover, the fruit juices showed greater antibacterial potential than fruit ethanol extracts. In contrast, the growth of the different yeast cultures exhibited little (*Trichosporon cutaneum, Saccharomyces cerevisiae* and *Kluyveromyces marxianus var. lactis*) or no sensitivity to the fruit extracts and juices. Other studies also identified Gram-positive pathogenic bacteria (i.e., *Staphylococcus aureus* ATTC 6538, *Enterobacter faecalis* ATCC 29212, *Listeria monocytogenes* ATCC 19115) as more sensitive to VO fruit components, than Gram-negative bacteria [25]. Moreover, the authors showed that the VO juice enriched with phenolic compounds showed better antimicrobial properties than fresh juice contained other non-phenolic components. Both tested juices did not influence the growth of *Escherichia coli* ATCC 10536 and 8739, *Pseudomonas aeruginosa* ATCC 15442 and 24755, *Enterobacter cloacae* ATCC 13047, *Salmonella typhimurium* ATCC 14028, *Salmonella enteritidis* ATCC 13076, and the yeast *Candida albicans* ATCC 10231. It could be suspected that the observed antibacterial action of VO fruit juices involves the influence of chlorogenic acid and procyanidins on the bacterial cell wall and membrane rigidity, permeability, or integrity influencing metabolism [85,86]. The antimicrobial activity of VO fruit extracts depended on the form of the fruit (fresh or dried) and the type of extraction solvent (hot and cold water, or hot and cold ethanol) [19]. Among the studied extracts the best antibacterial activity was obtained with hot water extracted from dried fruits. It inhibited the growth of *Staphylococcus aureus* ATCC 25923, *Staphylococcus epidermidis* ATCC 12228, and *Streptococcus pyogenes* ATCC 19615. In comparison, both cold water extracts, hot ethanol extract from fresh fruits, and cold ethanol extract from dried fruits showed no activity. Opposite relationships were observed by Eryilmaz and co-workers [16], because the aqueous extracts obtained from stem, flower, and leaf of VO did not show antibacterial and antifungal activities. Only the aqueous extract of VO leaf showed weak antibacterial activity against *Klebsiella pneumoniae* RSKK 574. For comparison, ethanol extracts from VO leaf and steam showed activity against *Staphylococcus aureus* ATCC 25923 and ATCC 43300 (MRSA), from leaf and flower against *Escherichia coli* ATCC 25922, *Pseudomonas aeruginosa* ATCC 27853, and *Klebsiella pneumoniae* RSKK 574. 

Because of its antimicrobial properties, VO has been used as plant material for production of organic–inorganic nanobiocatalytic and antimicrobial agents, which further can be used in textile, biosensor, and biomedicine [87]. Methanol extract from dried fruits was used as an organic agent to form copper(II) ions phosphate-based nanocrystals. Whereas VO extract did not show an effective antimicrobial activity up to 2000 μg/mL concentration, the nanoparticles displayed antimicrobial activity against studied microorganisms. The growth inhibition of *Escherichia coli* ATCC 35218, *Staphylococcus aureus* ATCC 25923, and *Salmonella typhimurium* ATCC 14028 was observed for minimal inhibitory concentration (MIC) values equal to 70 µg/mL dose of nanoparticles. The growth of Gram-positive bacteria *(Enterococcus faecium* ATCC 8459, *Enterobacter faecalis* ATCC 29212 and *Bacillus cereus* ATCC 11778) and fungi (*Candida albicans* ATCC10231, *Candida glabrata* ATCC 90030) was also inhibited with the MIC values from 1 to 7 µg/mL. Simultaneously, no effect was observed against *Pseudomonas aeruginosa* ATCC 27853. 

On the other hand, it was demonstrated that the growth of Gram-positive lactic acid bacteria (LAB), like *Lactobacillus rhamnosus* GG, *Lactobacillus plantarum* ŁOCK 0981, *Lactobacillus brevis* ŁOCK 0983, *Lactobacillus paracasei* ŁOCK 0985, *Lactobacillus delbrueckii* ŁOCK 0987 and *Lactobacillus plantarum* ŁOCK 0989, which are beneficial for human health and are the inhabitants of the gastrointestinal tract, were resistant to VO fruit components [25]. The observed effect is beneficial as it is known that alterations in composition and the diversity of gut microbiota can lead to inflammation and metabolic disorders. Therefore, the usage of beneficial intestinal microflora creates “colonization resistance” protecting the gut against freshly ingested microorganisms influencing host health [88]. Consequently, VO products enrichment with *Lactobacillus* strains could reveal their new pro-health properties. These results are in line with data showing that VO fruit juices (gilaburu) fermented with lactic acid bacteria (mainly *Lactobacillus plantarum*) may be considered as a functional food because of antimicrobial activity and probiotic potential [3]. Yetim et al. studied the gilaburu juice as a component of cemen paste (used in the production of dried-cured meat consumed in Turkey) with antimicrobial and colorant properties, however, it did not show a significant and positive influence on the studied product properties [89]. 

The antimicrobial VO properties were used to give functionality to fabrics. Woolen fabrics dyed with VO juice inhibited the growth of *Escherichia coli* by 3.19%, *Pseudomonas aeruginosa* by 0.77%, and *Candida albicans* by 2.05% in comparison to undyed wool fabric [90]. In the dyeing of cotton fabrics, good antibacterial efficiency against the *Escherichia coli* and *Staphylococcus aureus* was obtained with the use of VO fruit juice while VO branch extract showed no such effect.

Additionally, the elevation of temperature to 100 °C during the dyeing process abolished juice activity against *Escherichia coli* probably because of the degradation of phenolic compounds [91]. The authors suggested that the observed antibacterial properties of VO juice can be related to the presence of the high amount of metal ions especially copper and zinc in addition to the phenolic compounds responsible for the antibacterial properties. 

### 5.3. Effect on Carbohydrates Metabolism

Chronic elevation of postprandial hyperglycemia manifests the irregularity in glucose metabolism related to type 2 diabetes mellitus (T2DM). Because increased blood glucose concentration leads to oxidative stress generation, insulin resistance induction, and tissue damage, therefore there is a need for finding phytocompounds with hypoglycemic activity. Inhibition of carbohydrate- or oligosaccharide-hydrolyzing enzymes can be beneficial in lowering the postprandial glucose concentration. Among the digestive enzymes, the hydrolysis of internal 1,4-glycosidic linkages of starch to maltose, maltotriose, or dextrins is performed by α-amylase, whereas α-glucosidase catalyzes glucose release from disaccharides and oligosaccharides. A screening study with cell-free assay identified VO fruits acetone extract as able to inhibit pancreas α-amylase with IC_50_ between 0.97 and 2.19 mg/mL, depending on the type of assay performed [20]. Simultaneously, IC_50_ for the inhibition of α -glucosidase was equal to 4.05 mg/mL. 

On the other hand, the blood glucose level is strongly influenced by glucose transport and absorption in the small intestine by membrane-integrated transporters. An in vitro study demonstrated that phenolic-rich fraction obtained from VO fresh fruit juice at 50 µg/mL dose decreased the uptake of fluorescent glucose analogue 2-(N-(7-nitrobenz-2-oxa-1,3-diazol-4-yl)amino)-2-deoxyglucose (2-NBDG) by human adenocarcinoma Caco-2 cells by almost 20% [21]. Methanol-acetone extract from VO fruit pomace had lower efficacy and decreased 2-NBDG uptake by circa 10%. Glucose transporter 2 (GLUT2) is the main protein involved in intestinal bi-directional glucose transport across the enterocyte membrane. However, the further study demonstrated no influence of VO extract on *GLUT2* mRNA level [21]. That observation allowed authors to suppose that phenolic constituents might act as an inhibitor of GLUT2 protein rather, which in turn will decrease the absorption of glucose released from the meal. 

**Figure 2:**
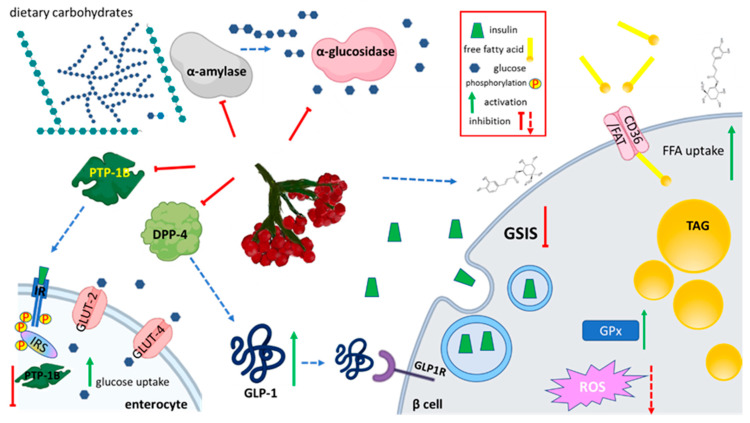
Proposed molecular mechanisms of anti-diabetic actions of *Viburnum opulus*; see the main text for more details. CD36/FAT—fatty acid translocase; DPP-4—dipeptidyl peptidase-4; FFA—free fatty acids; GLP-1—glucagon-like peptide-1; GLP1R—glucagon-like peptide-1 receptor; GLUT-2—glucose transporter 2; GLUT-4—glucose transporter 4; GPx—glutathione peroxidase; GSIS—glucose stimulated insulin secretion; IR—insulin receptor; IRS—insulin receptor substrate; PTP-1B—protein tyrosine phosphatase 1B; ROS—reactive oxygen species; TAG—triacylglycerols.

In the regulation of blood glucose level in the human body insulin is involved, which is secreted by pancreatic cells after the elevation of glucose concentration. The effect of VO fruit juice on pancreatic β-cells activity was also evaluated [22]. Cell-based studies demonstrated that VO fresh juice and juice enriched with phenolics at 75 and 50 µg/mL dose, respectively, decreased glucose-stimulated insulin secretion (GSIS) in mouse MIN-6 cells by 20–35% (Figure 2). Moreover, both preparations increased insulin secretion (by 10–15%) at low glucose concentration. These results suggested that VO could be involved in insulin resistance development, it may deepen insulin insufficiency, decrease glucose uptake by peripheral tissues, and elevate hyperglycemia. Authors proposed that observed in vitro GSIS inhibition resulted from the direct influence of extract constituents on cellular membranes—they observed the decrease of MIN-6 membrane fluidity and its hyperpolarization, which in turn inhibited insulin release. On the other hand, the corresponding dose of VO samples elevated secretion of glucagon-like peptide-1 (GLP-1) by enterocytic GLUTag cells in the presence of 20 mM glucose. That protein is an incretin hormone, which after binding to its receptor (GLP1R) stimulates insulin secretion from β-cells. Results demonstrated that VO samples increased the life time of GLP-1 peptide by direct inhibition of dipeptidyl peptidase-4 (DPP4) enzyme, which is responsible for the fast degradation of GLP-1 activity. In the presence of VO, the activity of DPP4 was decreased by 40%. Therefore, despite the potential side effect of VO on the GSIS process, the other extract properties could be helpful to obtain normalized glucose concentration. Still, it needs to be emphasized that VO samples intensified free fatty acids uptake and lipids accumulation in MIN-6 cells cultured in the presence of an elevated concentration of palmitic acid. These observations may contribute to the potentially lipotoxic effects of VO leading to β-cells dysfunction and death.

Another enzyme—protein tyrosine phosphatase 1B (PTP-1B)—is also involved in glucose cellular uptake regulation. This protein dephosphorylates insulin receptor (IR) and insulin receptor substrate IRS-1 disrupting the insulin signaling pathway. Thus, PTP-1B inhibitors are thought to increase the insulin sensitivity. A chemical-based study revealed that VO fruit acetone extract was a strong inhibitor of PTP-1B with IC_50_ equal to 0.15 mg/mL [20]. 

Compared to many in vitro studies on cell lines, there is only one in vivo study investigating the hypoglycemic activity of VO [92]. Studies with the Swiss albino mice with alloxane-induced diabetes showed that water extract obtained from air-dried and powdered leaves at 100 mg/kg dose had no effect in terms of lowering blood glucose [92]. It is worth noting that in the in vivo models, extracts of VO leaves were used instead of extracts of VO fruits, as in the in vitro studies. 

### 5.4. Effect on Lipid Metabolism 

Disturbance of homeostasis of lipid metabolism and lack of physical activity often leads to overweight, obesity, and type 2 diabetes. To approach the decrease of fatty acids absorption, inhibition of enzymes involved in fat digestion could be used. Pancreatic lipase splits triglycerides into absorbable monoacylglycerols and fatty acids in the intestinal lumen. It was demonstrated that acetone extract from dried VO fruits and isolated phenolic compounds inhibited the pancreatic lipase activity with IC_50_ equal to 11.5 and 3.3 mg/mL, respectively [23]. Moreover, isolated phenolics demonstrated synergistic action with orlistat, the known clinically approved lipase inhibitor. On the other hand, the juice obtained from fresh fruits was able to inhibit lipase activity by 50% at 262 mg/mL concentration, whereas IC_50_ for its isolated phenolics was equal to 55 mg/mL [24]. It can be concluded that extract obtained from dried plant material, which was more active against lipase, contained other bioactive compounds produced during the drying process of fruits. 

So far, there is no study demonstrating direct VO influence on weight gaining during in vivo studies, therefore the presented review focuses mainly on cell-based studies. It is known that dietary fatty acids are mainly absorbed and transported to the circulation from intestine using protein transporters, mainly protein known as cluster of differentiation 36/fatty acid translocase (CD36/FAT). VO fruit phenolics (50 µg/mL) decreased *CD36/FAT* mRNA expression level in Caco-2 cells treated with elevated concentration of palmitic acid [21]. Additionally, the uptake of free fatty acid fluorescent analogue TF2-C12 by enterocytes, as well as the accumulation and size of lipid droplets, was downregulated in cells by at least 10%. 

Obesity is characterized by the accumulation of excess fat in adipose tissue and results from enhanced development of preadipocytes into adipocytes, known as adipogenesis. Under in vitro conditions it manifests by the increased ability of adipocytes to accumulate triacylglycerol in cytosolic lipid droplets. The phenolic extract obtained from VO-dried fruits at 75 µg/mL reduced lipid content in differentiated mice 3T3-L1 adipocytes by 22% [23]. Research also revealed no influence of VO phenolics on adiponectin, the key regulator of insulin sensitivity. However, the downregulation of leptin, concentration of which is increased in obesity, was observed. Fresh juice and juice purified by solid-phase extraction enriched with phenolics also inhibited adipogenesis at doses equal to 100 and 25 µg/mL, respectively [24]. In this case, samples also decreased the uptake of free fatty acids (by circa 10%) and activated the lipolysis process (by 20%) leading to the breakdown of accumulated triacylglycerol to fatty acids and glycerol. Purified by the SPE method VO juice increased the expression of adiponectin mRNA and secreted protein (25%), and at the same time decreased leptin secretion by 20%. In both of the presented studies, the importance of phenolic compounds as inhibitors of adipogenesis is postulated.

**Figure 3:**
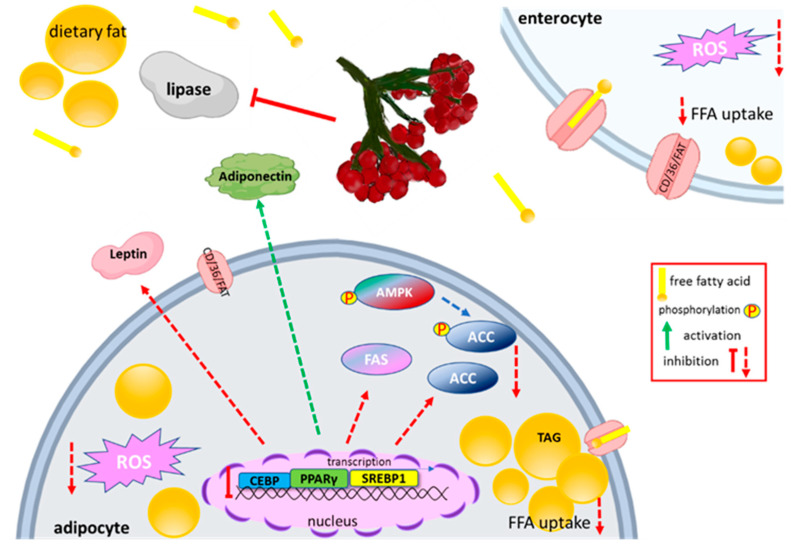
*Viburnum opulus* as adipogenesis process inhibitor; see the main text for more details. ACC—acetyl-CoA carboxylase; AMPK- AMP-activated protein kinase; CD36/FAT—fatty acid translocase; CEBP—CCAAT/enhancer-binding proteins; FAS—fatty acid synthase; FFA—free fatty acids; PPAR-γ—peroxisome proliferator-activated receptor gamma; ROS—reactive oxygen species; SREBP1—sterol regulatory element binding protein 1; TAG—triacylglycerols.

The study of Zakłos-Szyda et al. revealed the molecular mechanism involved in adipogenesis inhibition induced in cells incubated with VO [24]. It is known that the major adipogenesis regulator is peroxisome proliferator-activated receptor gamma (PPAR-γ), which belongs to the nuclear receptors family. After ligand binding to the PPAR-γ domain, the conformational change and switching of nuclear receptor corepressors to coactivators occur. After agonists binding, the PPAR-γ receptor connects with retinoid X receptor, and as a heterodimer translocates to peroxisome proliferator responsive element (PPRE) within the promoter of target genes responsible for adipogenesis and activates their transcription. Other important proteins cooperating with PPAR-γ receptor are sterol regulatory element binding protein 1c (SREBP-1c) and (C/EBP). VO samples impaired the differentiation of 3T3-L1 cells via downregulation of the key adipocyte differentiation regulatory genes mRNA and protein levels by at least 30% (Figure 3). Furthermore, the mRNA level of fatty acid synthase (*FAS)* involved in the synthesis of long-chain fatty acids, *CD36/FAT* transporter, and acetyl-CoA carboxylase (*ACC*) catalyzing the synthesis of the malonyl-CoA, was downregulated by 55.6 and 25%, respectively.

As given in Section 4, VO fruit is a very rich source of chlorogenic acid. Despite the fact that chlorogenic acid was identified as PPAR-γ nuclear receptor agonist [93], the presented study demonstrated that extract being a mixture of different phenolic compounds behaved rather as a PPAR-γ antagonist. A cell-based reporter gene assay confirmed this observation and indicated VO phenolic constituents as the potential antagonist of the PPAR-γ receptor. Performed docking simulation established the clogging of the entrance to the PPAR-γ binding pocket by procyanidins molecules, which in turn blocked chlorogenic acid from entering. That examination confirmed that the activity of a mixture of compounds may differ from observed for its separate constituents. Moreover, the regulation of lipid metabolism can be regulated by other signal transduction with AMP-activated protein kinase (AMPK) involvement [94]. This protein is known as a sensor of energy status that maintains cellular energy homeostasis. Among many proteins phosphorylated by AMPK is ACC. In this case ACC phosphorylation leads to this enzyme inactivation. Studies revealed that in the observed adipogenesis inhibition by VO juice elevation of p-ACC occurred. 

It is worth mentioning that despite presented cell-based results, the nuclear receptor ligand activity might be limited by its bioavailability and ligand intracellular uptake. As was mentioned before the main phenolic compound identified in VO juice was chlorogenic acid. Although it is extensively metabolized in humans with gut microbiota, it is believed that chlorogenic acid (as other phenolic aglycones) is absorbed in the small intestine by passive diffusion or with transporters, like P-glycoprotein carrier, and appears in the circulatory system as biotransformation products mediated by transferases as glucuronide, sulfate and methylated metabolites (where the last transformation improves polyphenols transportation through biological membranes) [95,96]. Thus nuclear receptors act as sensors of endobiotics and xenobiotics often through their biotransformation products. Nevertheless, in vitro studies showed that hepatic cells were able to stimulate chlorogenic acid derivatives uptake with organic anion transporting polypeptides (Oatps) [97]. Previous studies showed that phenolic compounds present in VO had binding affinity to human serum albumin, which is one of the factors determining drug bioavailability [22]. It is known that blood albumins are delivered to fluctuant microdomains (rafts) present on the plasma membrane of different cells and enter the cytoplasm through caveolin-dependent endocytosis [98]. Thus it could be assumed that phenolic compounds can be also delivered to rafts with albumins and regulate the activity of PPAR-γ, and other nuclear receptors, although further studies are needed to support this hypothesis. 

### 5.5. Anti-Inflammatory Potential

**Figure 4:**
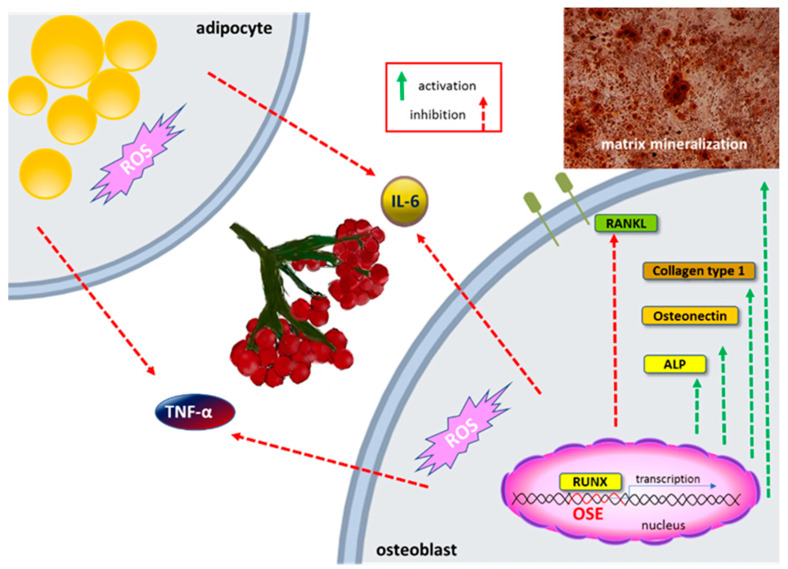
The proposed influence of *Viburnum opulus* on reduction of inflammatory response and regulation of osteogenesis process; see the main text for more details. ALP—alkaline phosphatase; IL-6—interleukin 6; OSE—osteoblast-specific cis-acting element; TNF-α—tumor necrosis factor α; RANKL—receptor activator of nuclear factor-kB ligand; RUNX—Runt-related transcription factor.

It is known that oxidative stress stimulates the secretion of inflammatory cytokines, which in turn leads to the development of different chronic diseases, like asthma, rheumatoid arthritis, multiple sclerosis, inflammatory bowel disease, or osteoporosis [99,100]. Among cytokines, the most related to obesity and insulin resistance are tumor necrosis factor-α (TNF-α) and interleukin-6 (IL-6). Research performed with phenolics isolated from fruits fresh juice demonstrated their ability to decrease secretion of TNF-α by 20% and IL-6 by 65% in differentiated adipocytes 3T3-L1 [24]. Both cytokines were also downregulated on mRNA and protein level in Saos-2 cells after treatment with fresh fruit juice and isolated phenolics (Figure 4) [25]. Stronger effect possessed phenolic fraction which at 25 µg/mL dose reduced IL-6 secretion by 55%, and TNF-α by 40%. In agreement with these data is experiments performed with ethanolic extract of VO bark, where 1 mg/mL dose decreased the secretion of TNF-α by 95% in lipopolysaccharide-stimulated human monocytic leukemia THP-1 cells [26]. 

Studies performed with amentoflavone, one of the flavonoids identified in VO leaves, exhibited that at 25 mg/mL dose it protected hippocampal neurons in the Kunming mice with epilepsy via a decrease of production of IL-6, TNF-α, IL-1β, and prostaglandin E2 [101]. In the observed mechanism the inhibition of nuclear factor-κB (NF-κB) occurred—the main regulator of inflammatory cytokines release. This protein is located as a dimer in the cytoplasm and functions as an inactive complex bound to its inhibitor I-κBα. Amentoflavone was demonstrated to abolishes the degradation of I-κBα, therefore reduced the activation of NF-κB. 

Despite anti-inflammatory potential observed in cell-based experiments, a study performed on the Sprague-Dawley rats treated with 100 and 200 mg/mL of water extract obtained from VO-dried leaves, had no anti-inflammatory effect on carrageenan paw edema in rats [6].

Other VO fruits constituents were demonstrated to be immunomodulators. The water-soluble polysaccharide fractions enhanced phagocytosis and the secretion of lysosomal enzymes with peritoneal macrophages [102]. Fractions were composed of galacturonic acid, galactose, arabinose, rhamnose, and mannose, whereas the most effective were acidic polysaccharides composed of β-1,4-linked galactopyranose residues of terminal and 2,5- and 3,5-substituted residues of α-arabinofuranose at a galactose to arabinose ratio of 3:1. 

### 5.6. Osteogenic Activity 

One of the unwanted symptoms related to obesity is chronic inflammation, which in turn induces the osteoclastogenesis process leading to bone tissue demineralization and osteoporosis [98]. It was reported that VO juice and isolated phenolics increased the mineralization of the extracellular matrix of human osteogenic Saos-2 cells (Figure 4) [25]. Stronger osteogenesis potential revealed purified phenolic extract, which at 25 µg/mL dose increased alkaline phosphatase activity by 35%. This enzyme is involved in matrix calcification and mineralized nodules formation. After cell treatment, the increase of mRNA level of the Runt-related transcription factor (*RUNX2*) was observed. RUNX2 is the major osteogenesis regulator, which binds to osteoblast specific cis-acting element (OSE) in the promoter region of the major osteoblast bone matrix protein genes and controls their expression [103]. As was shown osteogenic differentiation markers related to matrix mineralization, such as collagen type 1 and osteonectin, were elevated at the transcription level in cells treated with phenolics isolated from VO. Purified phenolic extract also decreased the receptor activator of nuclear factor-kB ligand (*RANKL*) mRNA level, therefore preventing its binding with the receptor present in osteoclast, and osteoclastogenesis. 

### 5.7. Influence on Blood Vessels Activity

In the inflammation state and blood pressure regulation, angiotensin I-converting enzyme (ACE I) is also involved. This protein converts the angiotensin I into the potent vasoconstricting angiotensin II and degrades bradykinin, a potent vasodilator inhibitor [104]. Therefore inhibitors of ACE I play an important role in the regulation of blood pressure and fluid and electrolyte balance. Performed cell-free screening identified ethanolic extract of VO fruits as a weak inhibitor of ACE I, where dose 0.1 mg/mL decreased the enzyme activity by 5.1%. For comparison, ethanol extract from bark had no effect on ACE I activity. 

Bujor et al. reported that VO fruit extract showed vasorelaxant activity in phenylephrine precontracted rat aortic rings with concentration giving half-maximal relaxation equal to 6.31 μg/mL [32]. Moreover, VO at 71.02 μg/mL inhibited the activity of arginase enzyme by 50%. This hydrolyzes L-arginine to L-ornithine and urea and is implicated in the regulation of nitric oxide (NO) synthesis by limiting the availability of intracellular L-arginine for nitric oxide synthase (NOS), and attenuates smooth muscle relaxation. The authors proposed VO fruit extract usage as a remedy in diseases associated with endothelial dysfunction and impaired vasodilation.

Studies performed on human umbilical endothelial cells (HUVECs) demonstrated a lack of the toxic effect of VO fruit juice or purified fruit juice rich in phenolic compounds (100 and 25 μg/mL, respectively) on the metabolic activity of cells [25]. Samples had no effect on the secretion of vascular endothelial growth factor (VEGF), which is involved in angiogenesis and may promote the healing of injured tissue.

### 5.8. Effect on Urinary System and the Endometriosis

So far, the VO effect on urinary system is the most efficiently investigated. Nephrolithiasis, known also as urolithiasis, is a kidney stone disease that develops in the urinary tract. A study performed on the Wistar rats with sodium oxalate-induced urolithiasis demonstrated that lyophilized ethanol extract obtained from VO fruits and juice, administrated orally in dose 100 mg/mL, induced a diuretic effect and reduced the level of oxalate, the main constituent of the stones [30]. This was followed by elevation of kidney glutathione and total thiols parameters, as well as the reduction of lipid peroxidation and thiobarbituric acid reactive substances (TBARS). Similar studies were conducted with other ethanol-acetic, methanol, and n-hexane extracts obtained from VO fruits. They showed less potent anti-urolithiatic properties than ethanolic extract. Moreover, a histopathological study identified the most irregularly shaped calculus fragments in kidney tissue after the animals were treated with ethanol-acetic preparation. Erdem and co-workers evaluated the effects of VO fruit ethanol extract on nephrolithiasis in the Spraque-Dawley rats model [31]. The authors showed that oral administration of both 0.5 and 1 mL of the extract at a concentration of 10 mg/mL to rats for five days a week during a one-month period had a beneficial effect on the urinary system. VO fruit extract increased the urine volume and urine citrate levels, decreased urine cystine and oxalate levels, and lowered the crystal deposits in kidney tissue, and also prevented oxidant damage and crystal formation in kidney tissue. Other studies also demonstrated VO as useful in the treatment of mild to moderate level hypocitraturic stone disease [105]. Juice obtained from fruit kept in brine water for a month was used as a source of bioactive components, which has a comparable level of citrate to lemon juice. Authors suggested VO juice usage as an alternative pharmaceutical treatment method in this disease, however, there were no details presented about the used treatment. 

Recent retrospective research performed on human subjects presented a positive effect of VO on patients with distal ureteral stones with a diameter of <10 mm [106]. It was evaluated that after treatment with diclofenac and 1000 mg of VO extract (peroral 3 x 2 on-demand) the elapsed time to stone expulsion was significantly shorter than in the group treated with diclofenac only. Additionally, in VO group the rate of stone expulsion was significantly higher and the need for additional treatment (ureteroscopy or extracorporeal shock wave lithotripsy) and the analgesic requirement was lower. Despite these positive results, it needs to be mentioned that among 53 patients involved in the trial and assigned to VO treatment, five patients developed dyspeptic complaints with upper abdominal pain. There was also evidence about the induction of severe abdominal pain and acute pancreatitis in a 38-years old male patient with urolithiasis, who drank 2 glasses of VO extract water daily for 4 days [107]. 

In addition to the above mentioned VO therapeutic potential against urolithiasis, the inhibition of chemical painful stimuli and antinociceptive potential was also demonstrated [6]. Water extract obtained from VO air-dried and powdered leaves at 100 and 200 mg/mL doses inhibited the acetic acid-inducted abdominal stretching response in the Swiss albino mice by 57% and 63%, respectively. The Tail-flick test with radiant heat as stimuli exhibited an antinociceptive effect similar to that of the morphine group at the 90th min, whereas the maximal effect in pain reduction (62%) was obtained for 200 mg/kg dose after 150th min. 

Because of the uterine relaxant and antispasmodic properties, VO bark is used as a traditional medicine in the treatment of premenstrual syndrome to reduce menstrual fluid volume and decrease the pain associated with uterine contractions [55,108]. Yet, there are no trials evaluating its efficacy in this regard. Nonetheless, a recent study demonstrated VO fruit potential in the treatment of endometriosis manifesting by the presence of endometrial tissue outside the uterine cavity with commonly occurring pelvic pain [109]. In vivo studies performed on the Sprague Dawley rats with induced endometriosis by uterine tissue auto- transplantation were treated with extracts obtained from air-dried fruits at dose 100 mg/kg for 28 days. Among the studied samples the ethyl acetate and methanol extracts decreased the sizes of endometric implants and their adhesion the most effectively. The decrease of inflammation was also observed—after VO treatment the TNF-α, VEGF, and IL-6 levels were reduced. Since both extracts were rich with chlorogenic acid (its concentration in methanol extract was three-folds higher than after extraction with ethyl acetate), authors attributed the observed activity to chlorogenic acid. 

### 5.9. Anti-Cancer Activity

Because of the lower adverse side effects in the prevention and therapy of cancer the phytochemicals are gaining more attention. So far there are only a few studies proving the anticancer properties of VO with animal subjects. A study performed by Ulger et al. demonstrated the protective effect of VO juice against 1,2-dimethylhydrazine (DMH)-induced colon cancer in the Balb-c mice [110]. Juice administration after tumor induction (for 18 weeks) and simultaneously with the chemical inducer (for 30 weeks) reduced tumor lesion and sufficiently downregulated the progress of established tumors in both cases. However, treatment with juice was not able to prevent the induction of colon cancer. In another study, juice of VO fresh fruits (1, 2, and 4 mg/kg) displayed antitumor activity in the Balb/c mice with intraperitoneal inoculation of Ehrlich ascites carcinoma (EAC) cells [111]. The Ehrlich tumor belongs to a rapidly growing and aggressive carcinoma. Histopathological findings demonstrated that in animals treated with the highest dose of juice the liver, colon, small bowel, and kidney had no pathological changes, therefore the metastasis was not observed. Because VO treatment elevated SOD and CAT activities authors associated the observed effect with the antioxidant potential of juice constituents and cytotoxic activity against EAC cells, which was proved in in vitro study. 

Although some anticancer effects have been attributed to VO, molecular mechanisms underlying these effects are partially explained with cell-based assays. It is known that migration of cells could be implicated in cancer metastasis and tumor formation. VO fruit preparations at concentrations without cytotoxic effect, decreased migration of human cervical cancer HeLa and breast cancer MCF-7 cell lines [112]. The most active migration inhibitor was the phenolic-rich fraction obtained from fresh juice, which reduced scratch area by nearly 70% in comparison to control cells. Other studies confirmed that commercially prepared gilaburu juice (80 µl/mL) revealed a cytotoxic effect against HeLa and Caco-2 cells, whereas no inhibition of metabolic activity or tube formation were observed in normal HUVEC cells [28]. Preparation obtained from defatted VO berry pomace after extraction with pressurized ethanol decreased the proliferation of human colon adenocarcinoma HT-29 cells (IC_50_ = 0.39 mg/mL) without toxic effect on Caco-2 cells [49]. Furthermore, the cytotoxic activity of phenolic compounds obtained from VO-dried leaves was assessed. Extracts at doses 400–500 µg/mL reduced the metabolic activity of human colorectal adenocarcinoma HT29 and SW480 cells more efficiently than in normal colon CCD841CoN cells [27]. Unfortunately, the authors did not show any studies explaining the presented observations differentiating the response of normal and neoplastic cells. Moreover, despite the cytoprotective activity of low doses of VO fruit extracts, the elevated concentration allowed to efficiently induce apoptosis in different cancer cellular models, such as Caco-2, MIN-6, HeLa, and MCF-7 cells [21,22,112]. The authors concluded that the mechanism of observed apoptosis induction occurred with the intrinsic mitochondrial pathway involvement and further activation of downstream caspases-3/7. The apoptotic type of death is known to be less destructive to neighboring cells than necrosis. The engulfment of apoptotic bodies by neighboring cells prevents the release of active enzymes or inflammatory signals from dying cells, which limits the damage of adjacent tissue. Therefore, the proapoptotic potential of VO shows that it could be an active food ingredient in products designed for the prevention of cancer. 

### 5.10. Cytoprotective Properties 

*Cytoprotection* is a process by which chemical compounds provide protection to cells against harmful agents. To understand the cytoprotective potential of phytocompounds there can be used cell-based assays or animal models. Because of the high content of phenolic compounds, as well as other components with antioxidative potential, VO revealed properties to neutralize or scavenge free radicals via chemical interaction with active molecules. 

VO fruit juice, as well as isolated phenolic compounds (50–75 µg/mL), showed protective activity against oxidative stress generated by *t*-BOOH in human hepatoma HepG2, insulin secreting β-TC3 and MIN-6 cells, as well as partially restored their metabolic activity [20,22]. Other study identified neuroprotective activity of VO leaf methanol extract as inhibitor of acetylcholinesterase (AChE), which via degradation of acetylcholine is responsible for the development of Alzheimer’s disease [75]. As it was demonstrated with cell-free assay, the 100 µg/mL dose inhibited AChE by almost 88%. 

So far, there are some in vivo studies about antioxidant enzymes regulation by VO components. Zayachkivska et al. investigated the influence of proanthocyanidins isolated from air-dried VO fruits on gastrointestinal mucosal damage induced by water immersion and resistant stress in the Wistar albino rats [83]. The results showed that proanthocyanidins doses 25–75 mg/kg applied orally increased the activity of SOD, CAT, and GPx in gastric mucosa, as well as reduced the damage of the tissue in stressed animals, therefore can be used as gastroduodeno protective agents. In another study, the effect of VO-dried fruit components against ischemia-reperfusion induced oxidative stress during the lung transplantation in the Wistar rats was investigated [84]. In that study methanol extract obtained from air-dried and powdered fruits was administered intra peritoneally in a dose of 200 mg/kg to recipient and donor. In group with induced oxidative stress the SOD, CAT, GPx activities and total glutathione level were markedly lower, whereas VO treatment compensated this effect. Malondialdehyde level in the lung tissue, which corresponds to lipid peroxidation of cellular membranes, as well as level of plasma protein carbonyl, were reduced in rats treated with VO fruit extract. These findings indicated that VO can be used as therapeutic against oxidative stress during lung transplantation. 

Sarıözkan et al. suggested that simultaneous consumption of VO-dried fruit water extract (100 mg/mL weekly) prevented and reduced the structural and functional damages in reproductive organs, tissues, and cells in the Wistar albino rats caused by taxane class chemotherapeutics, such as docetaxel and paclitaxel [113]. Mechanistic studies showed that elevation of SOD, CAT, and GPx activity occurred in testis and epididymis of group co-treated with VO, as well as the decrease of lipid peroxidation. VO was shown as an agent downregulating increments in germ cell apoptosis and testicular histo- and cytopathological damages in animals treated with paclitaxel. 

Altun et al. studied the effect of water extract of VO leaf on carbon tetrachloride (CCl_4_)-induced hepatotoxicity in the Sprague-Dawley rats [92]. After animals treated with extract dose of 100 mg/kg for 7 days the level of proteins associated with liver damage, like aspartate aminotransferase, alanine aminotransferase, alkaline phosphatase, and bilirubin, was slightly downregulated. In addition the LD_50_ (lethal dose) of the extract was determined as 5.447 g/kg. 

Despite the above mentioned cytoprotective mechanisms, the integrity of genomic DNA is another important biomarker of redox status and the health of the cells. Recent in vitro studies indicated that juice isolated from VO fruit and pomace worked as protectants against DNA damage induced in human adenocarcinoma Caco-2 cells by methylnitronitrosoguanidine (MNNG) or hydrogen peroxide [21]. Studies revealed that all samples (at dose 50–75 µg/mL) induced DNA repairs more efficiently after cells exposition to hydrogen peroxide than to MNNG. Fresh juice and its isolated phenolic compounds (100 and 25 µg/mL, respectively) also revealed positive influence on DNA repairs after damage induced by MNNG in Saos-2 cells [25]. 

## 6. Conclusions 

A rich and diverse chemical composition of VO makes it a valuable plant with different biological effects as a food ingredient and a folk medicine. Currently, evidence on the bioactive components of VO, in particular phenolic compounds and iridoids, and their biological activity is growing. The present review updates information on general properties of VO and focuses on its chemical composition, and biological properties, including antimicrobial, anti-oxidative, anti-inflammatory, cytoprotective, anti-cancer, anti-obesity, and anti-diabetic activities. Additionally, the review shows that most of the researches so far have been carried out on fruit, while only a few data concern other morphological parts of the plant such as bark, steam, leaf, or flower. Many mechanisms performed mainly with the cell-based assays have been proposed to explain how VO compounds exert biological protective effects. On the other hand, the molecular mechanisms underlying these effects under in vivo condition are poorly known. VO treatment was shown to induce diuretic effect, decrease inflammatory response during endometriosis, reduce the formation of ureteral stones and pain associated with uterine contractions. However, the demonstrated side effects of VO use indicate the need to intensify research on the bioavailability of VO phytochemicals introduced into the body, both in the form of food and various preparations. These results will be important for careful consideration of the use of VO as a functional ingredient, despite being a promising source of health-beneficial phytochemicals. Thus, the mechanisms responsible for the mode of action of VO are worth further research in both basic research and clinical medicine.

## Figures and Tables

**Table 1:** Content (mg/100 g fresh weight) of individual organic acids in *Viburnum opulus* fruits.

Organic Acid	Content (mg/100 g)	References
Malic acid	578–2090	[9]
Citric acid	270–1630	
Quinic acid	52–346	
Shikimic acid	0–5	
Malic acid	1083	[50]
Oxalic acid	81	
Citric acid	39	
Tartaric acid	120–144	[40]
Malic acid	110–135	
Fumaric acid	10–18	
Succinic acid	4–6	
Tartaric acid	113–135	[41]
Malic acid	108–122	
Fumaric acid	9–18	
Succinic acid	3–7	
Tartaric acid	124–141	[2]
Malic acid	121–137	
Fumaric acid	15–16	
Succinic acid	5	
Acetic acid	2.6–3.2	

**Table 2:** Concentrations of total phenolics, flavonoids, anthocyanins, and proanthocyanidins determined by spectrophotometric methods in different morphological parts of *Viburnum opulus.*

Part of Plant	Total Phenolics	Total Flavonoids	Total Anthocyanins	Total Proanthocyanidins	Reference
Fruit	753–1460 ^a^	n.a.	23–45 ^f^	n.a.	[33]
	403–733 ^a^	n.a.	n.a.	201–528 ^h^	[9]
	325.4 ^a^	n.a.	65	n.a.	[5]
	680–831 ^a^	314–489 ^c^	n.a.	n.a.	[8]
	621–987 ^a^	202–318 ^c^	15–51 ^g^	n.a.	[40]
	703–911 ^a^	187–267 ^c^	6–48 ^g^	n.a.	[41]
	668–856 ^a^	n.a.	27–53 ^g^	n.a.	[43]
	549–1105 ^a^	n.a.	n.a.	n.a.	[44]
	3730 ^b^	2010 ^d^	n.a.	520 ^i^	[34]
	4450 ^b^	n.a.	n.a.	n.a.	[61]
Seed	1231.0 ^a^	1032 ^e^	-	n.a.	[48]
Bark	3980 ^b^	2250 ^d^	-	1030 ^i^	[34]
Flower	3510 ^b^	1670 ^d^	-	220 ^i^	[34]

^a^ Expressed as mg of gallic acid equivalents/100 g of fresh weight; ^b^ expressed as mg of gallic acid equivalents/100 g of dry weight; ^c^ expressed as mg of rutin equivalents/100 g of dry weight; ^d^ expressed as mg of (+)-catechin equivalents/100 g of dry weight; ^e^ expressed as mg of (+)-catechin equivalents/100 g of fresh weight; ^f^ expressed as mg of cyanidin-3-glucoside equivalents/100 g of fresh weight; ^g^ expressed as mg of cyanidin-3-rutinoside equivalents/100 g of fresh weight; ^h^ expressed as mg of (+)-cyanidinin equivalents/100 g of fresh weight; ^i^ expressed as mg of (+)-cyanidin equivalents/100 g of dry weight; n.a.—not analyzed.

**Table 3:** Phenolic compounds identified in *Viburnum opulus* fruits.

Phenolic Compound		Content (mg/100 g of Fresh Weight)	References
Hydroxybenzoic acids	Gallic acid	10.82–11.82	[2]
	Vanillic acid	2.25–2.21	[2]
	Syringic acid	2.47–3.03	[2]
Hydroxycinnamic acids	Chlorogenic acid	2.95–4.43	[2]
		250–580	[9]
		203.7	[4]
	Caffeic acid	2.63–3.84	[2]
	Coumaric acid	1.40–1.73	[2]
	Ferulic acid	4.50–5.59	[2]
	Protocatechuic acid	2.09–3.63	[2]
Flavanols	Catechin	28.50–35.20	[2]
		29.04	[4]
	Epicatechin	2.69	[4]
	Procyanidin	8.28	[4]
Flavonols	Quercetin	0.61–0.83	[2]
	Quercetin 3-rutinoside	1.78–2.21	[2]
		0.9–5.2	[9]
		3.69	[4]
	Quercetin 3-sambubioside	2.0–10.6	[9]
	Quercetin 3-glucoside	0.1–3.4	[9]
		2.61	[4]
	Quercetin 3-rhamnoside	0.3–2.0	[9]
		1.01	[4]
	Quercetin 3-xyloside	0.34	[4]
	Quercetin 3-arabinoside	4.16	[4]
	Isorhamnetin 3-sambubioside	0.3–3.0	[9]
	Isorhamnetin 3-rutinoside	0–0.6	[9]
Anthocyanins	Cyanidin + 2 hexose + pentose	0–0.36	[9]
	Cyanidin + 2 pentose + hexose	0–2.11	[9]
	Cyanidin + 2 hexose	0–0.50	[9]
	Cyanidin + 2 pentose + hexose	0–0.13	[9]
	Cyanidin 3-arabinosyl-glucoside	0–10.42	[9]
	Unidentified cyanidin glycoside	0–0.71	[9]
	Cyanidin 3-xylosyl-rutinoside	0–19.87	[9]
	Cyanidin 3-sambubioside	0–0.87	[9]
	Cyanidin 3-glucoside	0.12–12.06	[9]
		7.23	[4]
	Cyanidin 3-rutinoside	0–6.39	[9]
		0.99	[4]

**Table 4:** Phenolic compounds identified in *Viburnum opulus* fruit juice.

Phenolic Compound		Content (mg/100 g)	References
Hydroxycinnamic acids	Chlorogenic acid	803.9 n.r.	[24] [1]
	Chlorogenic acid dimer	n.r.	[1]
	Neochlorogenic acid	0.7	[24]
	Cryptochlorogenic acid	0.4	[24]
	Caffeoylquinic acid derivatives	12.4	[24]
	Coumaroyl-quinic acid	n.r.	[1]
Flavanols	(+)-Catechin	65.7	[24]
		n.r.	[1]
	(-)-Epicatechin	13.5	[24]
		n.r.	[1]
	(Epi)catechin derivatives	18.3	[24]
	Gallocatechin gallate	3.1	[24]
	Procyanidin B1	75.9	[24]
	Procyanidin B2	19.9	[24]
		n.r.	[1]
	Procyanidin dimers	4.0	[24]
	Proanthocyanidin dimer monoglycoside	n.r.	[1]
	Procyanidin C1	3.3	[24]
	Procyanidin trimers	17.2	[24]
		n.r.	[1]
Flavonols	Quercetin 3-vicianoside	2.0	[24]
	Quercetin 3-rutinoside	1.6	[24]
		n.r.	[1]
	Quercetin 3-rhamnoside	0.7	[24]
	Quercetin hexose	n.r.	[1]
	Quercetin deoxyhexose	n.r.	[1]
	Quercetin hexose + pentose	n.r.	[1]
Anthocyanins	Cyanidin 3-sambubioside	9.3	[24]
	Cyanidin 3-glucoside	13.9	[24]
	Cyanidin 3-rutinoside	6.8	[24]

n.r.—not reported.

**Table 5:** Antioxidant capacities of *Viburnum opulus* leaf, branch, bark seed, and flower determined by single electron transfer (SET) or ET methods.

Type of Assay	Part of the Plant	Extraction Solvent	Antioxidant Activity Parameter	Reference
DPPH^●^ radical scavenging activity	Fruit	80% methanol with 2% HCl (*v/v*)	8.55–9.79 mg ascorbic acid equivalents/g of fruit FW	[8]
	Fruit	water	IC_50_ = 0.057 mg of extract/mL	[82]
	Fruit	80% acetone with 0.5% acetic acid (*v/v*)	IC_50_ = 0.057 mg of extract/mL	[32]
	Fruit	96% methanol	103.59 mg BHT equivalents/g of extract	[73]
	Fruit	water	96.74 mg BHT equivalents/g of extract	[73]
	Fruit flesh	methanol	EC_50_ = 24.56 mg/mg DPPH^●^	[59]
	Fruit pomace	acetone	121.8 mg Trolox equivalents/g of extract DW	[74]
	Fruit pomace	ethanol	106.9 mg Trolox equivalents/g of extract DW	[74]
	Fruit pomace	water	267.4 mg Trolox equivalents/g of extract DW	[74]
	Leaf	water	IC_50_ = 47.18 μg of extract/mL	[82]
	Branch	water	IC_50_ = 0.014 mg of extract/mL	[82]
	Seed	methanol	EC_50_ = 2.35 mg/mg DPPH^●^	[59]
ABTS^+●^ cation radical scavenging activity	Fruit	80% methanol with 2% HCl (*v/v*)	9.10–11.12 mg ascorbic acid equivalents/g of fruit FW	[8]
	Fruit	96% ethanol with 0.2% HCl (*v/v*)	7.05 mg ascorbic acid equivalents/g of frozen fruit	[37]
	Fruit	50% ethanol	643 μmol Trolox equivalents/g of fruit DW	[74]
	Fruit	70% ethanol	265.7 μmol Trolox equivalents/g of fruit DW	[76]
	Fruit	water	380.36 μmol Trolox equivalents/g of extract	[34]
	Fruit juice	-	31.95–42.38 μmol Trolox equivalents/g of juice	[53]
	Fruit pomace	acetone	376.8 μmol Trolox equivalents/g of extract DW	[74]
	Fruit pomace	ethanol	331.0 μmol Trolox equivalents/g of extract DW	[74]
	Fruit pomace	water	602.3 μmol Trolox equivalents/g of extract DW	[74]
	Fruit juice	-	70.17 μmol Trolox equivalents/mL of juice	[76]
	Bark	70% ethanol	402.1 μmol Trolox equivalents/g of bark DW	[34]
	Bark	water	1792.16 μmol Trolox equivalents/g of extract	[76]
	Flower	70% ethanol	161.8 μmol Trolox equivalents/g of flower DW	[34]
	Flower	water	475.95 μmol Trolox equivalents/g of extract	
FRAP—ferric reducing antioxidant power	Fruit	70% acetone with 0.5% acetic acid	21.02–34.90 μmol Trolox equivalents/g of fruit FW	[40]
	Fruit	(*v/v*)	23.41–32.70 μmol Trolox equivalents/g of fruit FW	[41]
	Fruit	70% acetone with 0.5% acetic acid	28.76–36.41 μmol Trolox equivalents/g of fruit FW	[43]
	Fruit	(*v/v*)	192.9 μmol Trolox equivalents/g of fruit DW	[76]
	Fruit	70% acetone with 0.5% acetic acid	311.34 μmol Trolox equivalents/g of extract	[34]
	Fruit	(*v/v*)	0.46 mmol FeSO_4_ equivalents/g of extract	[73]
	Fruit	70% ethanol	0.41 mmol FeSO_4_ equivalents/g of extract	[73]
	Fruit juice	water	64.35 μmol Trolox equivalents/mL of juice	[74]
	Fruit juice	96% methanol	32.33–35.94 μmol Trolox equivalents/g of juice	[53]
	Bark	water	234.7 μmol Trolox equivalents/g of fruit DW	[76]
	Bark	-	1160.30 μmol Trolox equivalents/g of extract	[34]
	Flower	-	136.5 μmol Trolox equivalents/g of fruit DW	[76]
	Flower	70% ethanol	463.91 μmol Trolox equivalents/g of extract	[34]
CUPRAC—cupric reducing antioxidant capacity	Fruit	96% methanol	208.87 mg ascorbic acid equivalents/g of extract	[73]
	Fruit	water	156.49 mg ascorbic acid equivalents/g of extract	[73]
ORAC—oxygen radical absorbance capacity	Fruit	70% ethanol	109.3 μmol Trolox equivalents/g of fruit DW	[76]
	Fruit	50% ethanol	1277 μmol Trolox equivalents/g of fruit DW	[74]
	Fruit juice	-	127.37–143.25 μmol Trolox equivalents/g of juice	[53]
	Fruit pomace	acetone	5750 μmol Trolox equivalents/g of extract DW	[74]
	Fruit pomace	ethanol	5320 μmol Trolox equivalents/g of extract DW	[74]
	Fruit pomace	water	8720 μmol Trolox equivalents/g of extract DW	[74]
	Bark	70% ethanol	1081.7 μmol Trolox equivalents/g of bark DW	[75]
	Bark	water	4386.19 μmol Trolox equivalents/g of extract	[34]
	Flower	70% ethanol	618.2 μmol Trolox equivalents/g of flower DW	[76]
	Flower	water	4283.41 μmol Trolox equivalents/g of extract	[34]
Nitric oxide scavenging activity	Fruit	50 mM phosphate buffer, pH 7.0	21.89–25.44% of inhibition for 25% fruit extract	[8]
Superoxide anion scavenging activity	Fruit	50 mM phosphate buffer, pH 7.0	25.13–28.50% of inhibition for 25% fruit extract	[8]
	Fruit	70% ethanol	897.7 μmol Trolox equivalents/g of fruit DW	[76]
	Leaf	water	IC_50_ = 8.3 mg of extract/mL	[63]
	Branch	water	IC_50_ = 3.7 mg of extract/mL	[63]
	Bark	70% ethanol	1154.4 μmol Trolox equivalents/g of bark DW	[76]
	Flower	70% ethanol	911.3 μmol Trolox equivalents/g of flower DW	[76]
Hydroxyl radical scavenging activity	Fruit	50 mM phosphate buffer, pH 7.0	19.40–23.98% of inhibition for 25% fruit extract	[8]
	Fruit	70% ethanol	100.5 μmol Trolox equivalents/g of fruit DW	[76]
	Bark	70% ethanol	59.1 μmol Trolox equivalents/g of bark DW	[76]
	Bark	water	191.42 μmol Trolox equivalents/g of extract	[34]
	Flower	70% ethanol	82.3 μmol Trolox equivalents/g of flower DW	[76]
	Flower	water	188.12 μmol Trolox equivalents/g of extract	[34]
DMPD scavenging activity	Fruit	96% methanol	52.55 mg Trolox equivalents/g of extract	[73]
	Fruit	water	55.00 mg Trolox equivalents/g of extract	[73]
Ferrous ion chelating capacity	Fruit	ethyl acetate	60.5% of inhibition at 2 mg of extract/mL	[75]
	Fruit	methanol	15.0% of inhibition at 2 mg of extract/mL	[75]
	Fruit	water	11.0% of inhibition at 2 mg of extract/mL	
	Leaf	ethyl acetate	21.0% of inhibition at 2 mg of extract/mL	
	Leaf	methanol	13.0% of inhibition at 2 mg of extract/mL	
	Leaf	water	0% of inhibition at 2 mg of extract/mL	
	Branch	ethyl acetate	6.5% of inhibition at 2 mg of extract/mL	
	Branch	methanol	23.0% of inhibition at 2 mg of extract/mL	
	Branch	water	22.5% of inhibition at 2 mg of extract/mL	
Lipid peroxidation in the rat liver homogenate	Fruit	50 mM phosphate buffer, pH 7.0	11.20–13.90% of inhibition for 25% fruit extract	[8]
Total antioxidant capacity	Fruit	96% methanol	56.89 mg ascorbic acid equivalents/g of extract	[73]
	Fruit	water	49.07 mg ascorbic acid equivalents/g of extract	[73]
CVA—cathode voltammetry	Bark	30% ethanol	K = 101.71 mL/g of extract	[46]
		70% ethanol	K = 181.52 mL/g of extract	[46]

DPPH—2,2-diphenyl-1-picrylhydrazyl; ABTS—2,2′-azino-bis(3-ethylbenzothiazoline-6-sulfonic acid); DMPD—*N*,*N*-dimethyl-p-phenylendiamine; IC_50_ = EC_50_ — the half maximal inhibitory concentration; K—antioxidant activity coefficient; FW—fresh weight; DW—dry weight.

## References

[1] Karaçelik A.A., Küçük M., Iskefiyeli Z., Aydemir S., De Smet S., Miserez B., Sandra P. (2015). Antioxidant components of *Viburnum opulus* L. determined by on-line HPLC–UV–ABTS radical scavenging and LC–UV–ESI-MS methods. Food Chem..

[2] Özrenk M., Gϋndoǧdu N., Kenskin N., Kaya T. (2011). Some physical and chemical characteristics of gilaburu (*Viburnum opulus* L.) fruits in Erzincan region. Iğdır Univ. J. Inst. Sci. Technol..

[3] Sagdic O., Ozturk I., Yapar N., Yetim H. (2014). Diversity and probiotic potentials of lactic acid bacteria isolated from gilaburu, a traditional Turkish fermented European cranberrybush (*Viburnum opulus* L.) fruit drink. Food Res. Int..

[4] Velioglu Y.S., Ekici L., Poyrazoglu E.S. (2006). Phenolic composition of European cranberrybush (*Viburnum opulus* L.) berries and astringency removal of its commercial juice. Int. J. Food Sci. Technol..

[5] Akbulut M., Calsir S., Marakoglu T., Coklar H. (2008). Chemical and technological properties of European cranberrybush (*Viburnum opulus* L.) fruits. Asian J. Chem..

[6] Altun M.L., Çitoǧlu G.S., Yilmaz B.S., Özbek H. (2009). Antinociceptive and anti-inflammatory activities of Viburnum opulus. Pharm. Biol..

[7] Konarska A., Domaciuk M. (2018). Differences in the fruit structure and the location and content of bioactive substances in *Viburnum opulus* and *Viburnum lantana* fruits. Protoplasma.

[8] Rop O., Reznicek V., Valsikova M., Jurikova T., Mlcek J., Kramarova D. (2010). Antioxidant properties of European cranberrybush fruit (*Viburnum opulus* var. edule). Molecules.

[9] Perova I.B., Zhogova A.A., Cherkashin A.V., Éller K.I., Ramenskaya G.V. (2014). Biologically active sunstances from European guelder berry fruits. Pharm. Chem. J..

[10] Baschali A., Tsakalidou E., Kyriacou A., Karavasiloglou N., Matalas A.L. (2017). Traditional low-alcoholic and non-alcoholic fermented beverages consumed in European countries: A neglected food group. Nutr. Res. Rev..

[11] Soylak A., Elci L., Saracoglu S., Divrikli U. (2002). Chemical analysis of fruit juice of European cranberrybush (*Viburnum opulus*) from Kayseri-Turkey. Asian J. Chem..

[12] Lachowicz S., Oszmianski J. (2018). The influence of addition of cranberrybush juice to pear juice on chemical composition and antioxidant properties. J. Food Sci. Technol..

[13] Al Ö., Ülger H., Eetekin T., Nisari M., Susar H., Ceylan D., Karatoprak G.Ş. (2017). The effect of gilaburu (*Viburnum opulus*) juice on Ehrlich ascites tumor (EAT) cell culture. Proceedings.

[14] Çemtekİn B., Kilinç E., Karabacak L., Dağtekİn T. (2019). Aa evaluationof guelder rose (*Viburnum opulus* L.) and hawthorn (*Crataegus monogyna*) concentrates as alternative antioxidant sources to BHT and nitrite in poultry meat model system. Sci. Pap. Ser. D. Anim. Sci..

[15] Česonienė L., Daubaras R., Kraujalytė V., Venskutonis P.R., Šarkinas A. (2014). Antimicrobial activity of *Viburnum opulus* fruit juices and extracts. J. Verbraucherschutz Leb..

[16] Erylimaz M., Ozbiligin S., Ergene B., Yilmaz S., Altun M.L., Saltan G. (2013). Antimicrobial activity of Turkish *Viburnum* species. Bangladesh J. Bot..

[17] Bubulica V.M., Anghel I., Grumezescu A.M., Saviuc C., Anghel G.A., Chifriuc M.C., Gheorghe I., Lazar V., Popescu A. (2012). In vitro evaluation of bactericidal and antibiofilm activity of *Lonicera tatarica* and *Viburnum opulus* plant extracts on *Staphylococcus strains*. Farmacia.

[18] Sagdic O., Aksoy A., Ozkan G. (2006). Evaluation of the antibacterial and antioxidant potentials of cranberry (gilaburu, *Viburnum opulus* L.) fruit extract. Acta Aliment..

[19] Turker H., Yıldırım A.B. (2015). Screening for antibacterial activity of some Turkish plants against fish pathogens: A possible alternative in the treatment of bacterial infections. Biotechnol. Biotechnol. Equip..

[20] Zakłos-Szyda M., Majewska I., Redzynia M., Koziołkiewicz M. (2015). Antidiabetic effect of polyphenolic extracts from selected edible plants as α-amylase, α-glucosidase and PTP1B inhibitors, and β pancreatic cells cytoprotective agents—A comparative study. Curr. Top. Med. Chem..

[21] Zakłos-Szyda M., Pawlik N., Polka D., Nowak A., Koziołkiewicz M., Podsędek A. (2019). *Viburnum opulus* fruit phenolic compounds as cytoprotective agents able to decrease free fatty acids and glucose uptake by Caco-2 cells. Antioxidants.

[22] Zakłos-Szyda M., Kowalska-Baron A., Pietrzyk N., Drzazga A. (2020). Evaluation of *Viburnum opulus* L. Fruit phenolics cytoprotective potential on insulinoma MIN6 cells relevant for diabetes mellitus and obesity. Antioxidants.

[23] Podsędek A., Zakłos-Szyda M., Polka D., Sosnowska D. (2020). Effects of *Viburnum opulus* fruit extracts on adipogenesis of 3T3-L1 cells and lipase activity. J. Funct. Foods.

[24] Zakłos-Szyda M., Pietrzyk N., Szustak M., Podsędek A. (2020). *Viburnum opulus* L. juice phenolics inhibit mouse 3T3-L1 cells adipogenesis and pancreatic lipase activity. Nutrients.

[25] Zakłos-Szyda M., Nowak A., Pietrzyk N., Podsędek A. (2020). *Viburnum opulus* L. juice phenolic compounds influence osteogenic differentiation in human osteosarcoma Saos-2 cells. Int. J. Mol. Sci..

[26] Kalinkevich K., Karandashov V.E., Ptitsyn L.R. (2014). In vitro study of the anti-inflammatory activity of some medicinal and edible plants growing in Russia. Russ. J. Bioorganic Chem..

[27] Chojnacka K., Owczarek K., Fichna J., Sosnowska D., Lewandowska U. (2019). Wpływ ekstraktów z liści kaliny koralowej (*Viburnum opulus* L.) na wzrost ludzkich komórek jelita. Post Fitoter.

[28] Kopral A.T. (2019). In vitro evaluation of gilaburu (*Viburnum opulus* L.) juice on different cell lines. Anadolu J. Educ. Sci. Int..

[29] Ucar T.A., Yildirim A.B., Karakas F.P. (2012). Antibacterial and antitumor activities of some wild fruits grown in Turkey. Biotechnol. Biotechnol. Equip..

[30] Ilhan M., Ergene B., Süntar I., Özbilgin S., Çitoʇlu G.S., Demirel M.A., Keleş H., Altun L., Akkol E.K. (2014). Preclinical evaluation of antiurolithiatic activity of *Viburnum opulus* L. On sodium oxalate-induced urolithiasis rat model. Evidence-based Complement. Altern. Med..

[31] Erdem G., Kesik V., Honca T., Ozcan A., Uguz S., Akgvl E.O., Aykutlug O., Alp B.F., Korkmazer N., Saldir M. (2016). Antinephrolithiatic activity of *Persea americana* (avocado) and *Viburnum opulus* (guelder rose) against ethylene glycol-induced nephrolithiasis in rats. Afr. J. Tradit. Complement. Altern. Med..

[32] Bujor A., Miron A., Luca S.V., Skalicka-Wozniak K., Silion M., Ancuceanu R., Dinu M., Girard C., Demougeot C., Totoson P. (2019). Metabolite profiling, arginase inhibition and vasorelaxant activity of *Cornus mas*, *Sorbus aucuparia* and *Viburnum opulus* fruit extracts. Food Chem. Toxicol..

[33] Česoniene L., Daubaras R., Vencloviene J., Viškelis P. (2010). Biochemical and agro-biological diversity of *Viburnum opulus* genotypes. Cent. Eur. J. Biol..

[34] Polka D., Podsedek A. (2019). Phenolics composition and antioxidant capacity of guelder rose fruit, flower and bark extracts. Biotechnol. Food Sci..

[35] Rychlińska I. (2008). Sterols and triterpenes in *Viburnum opulus* L. leaves. Herba Pol..

[36] Zarifikhosroshahi M., Tugba Z., Kafkas E., Okatan V. (2020). Variation in volatile and fatty acid contents among *Viburnum opulus* L. fruits growing different locations. Sci. Hortic..

[37] Moldovan B., Luminiţa D., Chişbora C., Cimpoiu C. (2012). Degradation kinetics of anthocyanins from European cranberrybush (*Viburnum opulus* L.) fruit extracts. effects of temperature, pH and storage solvent. Molecules.

[38] Kraujalyte V., Venskutonis P.R., Pukalskas A., Cesoniene L., Daubaras R. (2013). Antioxidant properties and polyphenolic compositions of fruits from different European cranberrybush (*Viburnum opulus* L.) genotypes. Food Chem..

[39] Eriksson O., Ehrln J. (1991). Phenological variation in fruit characteristics in vertebrate-dispersed plants. Oecologia.

[40] Ersoy N., Ercisli S., Gundogdu M. (2017). Evaluation of European cranberrybush (*Viburnum opulus* L.) genotypes for agro-morphological, biochemical and bioactive characteristics in Turkey. Folia Hortic..

[41] Ersoy N., Ercisli S., Akin M., Gundogdu M., Colak A.M., Ben Ayed R. (2018). Agro-morphological and biochemical characteristics of European cranberrybush (*Viburnum opulus* L.). Compt. Rend. Acad. Bulg. Sci..

[42] Kalyoncu I.H., Ersoy N., Elidemir A.Y., Korali M.E. (2013). Some physico-chemical characteristics and mineral contents of gilaburu (*Viburnum opulus* L.) fruits in Turkey. Int. J. Agric. Biosyst. Eng..

[43] Ozkan G., Ercisli S., Ibrahim H., Gulce S. (2020). Diversity on fruits of wild grown European cranberrybush from coruh valley in Turkey. Erwerbs-Obstbau.

[44] Ozrenk K., Ilhan G., Sagbas H.I., Karatas N., Ercisli S., Colak A.M. (2020). Characterization of European cranberrybush (*Viburnum opulus* L.) genetic resources in Turkey. Sci. Hortic..

[45] Moskalets T.Z., Moskalets V., Vovkohon A.H., Knyazyuk O.V. (2019). Fruits of new selection forms and varieties of snowball tree for manufacture of products of therapeutic and prophylactic purpose. Regul. Mech. Biosyst.

[46] Andreeva T.I., Komarova E.N., Yosubov M.S., Korotkova E. (2004). Antioxidant activity of cranberry tree (*Viburnum opulus* L.) bark extract. Pharm. Chem. J..

[47] Taşkin O., Asik B.B., Izli N. (2019). Mineral content of leaves, stalks and fruits of European cranberrybush plant (*Viburnum opulus* L.). KSÜ Tarım Doğa Derg..

[48] Turek S., Cisowski W. (2007). Free and chemically bonded phenolic acids in barks of *Viburnum opulus* L. and *Sambucus nigra* L.. Acta Pol. Pharm. Drug Res..

[49] Dienaite L., Pukalskiene M., Pereira C.V., Matias A.A., Venskutonis P.R. (2020). Valorization of European cranberry bush (*Viburnum opulus* L.) berry pomace extracts isolated with pressurized ethanol and water by assessing their phytochemical composition, antioxidant, and antiproliferative activities. Foods.

[50] Cam M., Hisil Y., Kuscu A. (2007). Organic acid, phenolic content and antioxidant capacity of fruit flesh and seedof *Viburnum opulus*. Chem. Nat. Compd..

[51] Yunusova S.G., Karimova A.R., Tsyrlina E.M., Yunusov M.S., Denisenko O.N. (2004). Change on storage of biological activity of *Viburnum opulus* seed components. Chem. Nat. Compd..

[52] Yilmaz N., Beyhan Ö., Gerçekçioğlu R., Kalayci Z. (2011). Determination of fatty acid composition in seed oils of some important berry species and genotypes grown in Tokat Province of Turkey. Afr. J. Biotechnol..

[53] Kraujalyte V., Leitner E., Rimantas P. (2012). Chemical and sensory characterisation of aroma of *Viburnum opulus* fruits by solid phase microextraction-gas chromatography—Olfactometry. Food Chem..

[54] Sönmezdağ A.S., Sevindik O., Kelebek H., Selli S. (2017). Aroma compounds of non-alcoholic fermented beverage: Gilaburu juice. EuroBiotech J..

[55] Yilmaztekin M., Sislioglu K. (2015). Changes in volatile compounds and some physicochemical properties of European cranberrybush (*Viburnum opulus* L.) during ripening through traditional fermentation. J. Food Sci..

[56] Udensi U.K., Tchounwou P.B. (2017). Potassium homeostasis, oxidative stress, and human disease. Int. J. Clin. Exp. Physiol..

[57] Yang C.S., Ho C., Zhang J., Wan X., Zhang K., Lim J. (2018). Antioxidants: Differing meanings in food science and health science. J. Agric. Food Chem..

[58] Nimse S.B., Pal D. (2015). Free radicals, natural antioxidants, and their reaction mechanisms. RSC Adv..

[59] Çam M., Hişil Y. (2007). Comparison of chemical characteristics of fresh and pasteurised juice of gilaburu (*Viburnum opulus* L.). Acta Aliment..

[60] Yang B., Ahotupa M., Maatta P., Kallio H. (2017). Composition and antioxidative activities of supercritical CO_2_ -extracted oils from seeds and soft parts of northern berries. Food Res. Int..

[61] Ignat I., Volf I., Popa V.I. (2011). A critical review of methods for characterisation of polyphenolic compounds in fruits and vegetables. Food Chem..

[62] Cory H., Passarelli S., Szeto J., Tamez M., Mattei J. (2018). The role of polyphenols in human health and food systems: A mini-review. Front. Nutr..

[63] Altun M.L., Yilmaz B.S. (2007). HPLC method for the analysis of salicin and chlorogenic acid from *Viburnum opulus* and *V. lantana*. Chem. Nat. Compd..

[64] Danielewski M., Matuszewska A., Nowak B., Kucharska A.Z., Sozanski T. (2020). The effects of natural iridoids and anthocyanins on selected parameters of liver and cardiovascular system functions. Oxid. Med. Cell. Longev..

[65] Sánchez-Marzo N., Lozano-Sánchez J., de la Luz Cádiz-Gurrea M., Herranz-López M., Micol V., Segura-Carretero A. (2019). Relationships between chemical structure and antioxidant activity of isolated phytocompounds from *Lemon verbena*. Antioxidants.

[66] Stępień A., Aebisher D., Bartusik-Aebisher D. (2018). Anticancer properties of *Viburnum*. Eur. J. Clin. Exp. Med..

[67] Bock K., Rosendal S., Bent J., Nielsen J., Norm V. (1978). Iridoid allosides from *Viburnum opulus*. Phytochemistry.

[68] Hussain T., Tan B., Yin Y., Blachier F., Tossou M.C.B., Rahu N. (2016). Oxidative stress and inflammation: What polyphenols can do for us. Oxid. Med. Cell. Longev..

[69] Lourenço S.C., Mold M., Alves V.D. (2019). Antioxidants of natural plant origins: From sources to food industry applications. Molecules.

[70] Pisoschi A.M., Pop A., Cimpeanu C., Predoi G. (2016). Antioxidant capacity determination in plants and plant-derived products: A review. Oxid. Med. Cell. Longev..

[71] Wu Q., Song R., Zhao L., Yun Z. (2019). Advances in cellular evaluation and standard of antioxidant activity. ChinaBiofilms.

[72] Zhang H., Yin M., Huang L., Wang J., Gong L., Liu J., Sun B. (2017). Evaluation of the cellular and animal models for the study of antioxidant activity: A review. J. Food Sci..

[73] Barak T.H., Celep E., Yesilada E. (2019). Influence of in vitro human digestion on the bioavailability of phenolic content and antioxidant activity of *Viburnum opulus* L. (European cranberry) fruit extracts. Ind. Crop. Prod..

[74] Kraujalis P., Kraujaliene V., Kazernaviˇ R., Venskutonis P.R. (2017). Supercritical carbon dioxide and pressurized liquid extraction of valuable ingredients from *Viburnum opulus* pomace and berries and evaluation of product characteristics. J. Supercrit. Fluid..

[75] Erdogan-Orhan I., Altun M.L., Sever-Yilmaz B., Saltan G. (2011). Anti-acetylcholinesterase and antioxidant assets of the major components (salicin, amentoflavone, and chlorogenic acid) and the extracts of *Viburnum opulus* and *Viburnum lantana* and their total phenol and flavonoid contents. J. Med. Food.

[76] Polka D., Podsędek A. (2019). Comparison of chemical composition and antioxidant capacity of fruit, flower and bark of *Viburnum opulus*. Plant Foods Hum. Nutr..

[77] Paşayeva L., Arslan A.K.K., Kararenk A.C. (2019). *Viburnum opulus* L. fruit extracts protect human neuroblastoma SH-SY5Y cells against hydrogen peroxide-induced cytotoxicity. Proceedings.

[78] Medina-Gomez G., Gray S., Vidal-Puig A. (2007). Adipogenesis and lipotoxicity: Role of peroxisome proliferator-activated receptor γ (PPARγ) and PPARγcoactivator-1 (PGC1). Public Health Nutr..

[79] Cumaoǧlu A., Rackova L., Stefek M., Kartal M., Maechler P., Karasu Ç. (2011). Effects of olive leaf polyphenols against H_2_O_2_ toxicity in insulin secreting β-cells. Acta Biochim. Pol..

[80] Ighodaro O.M., Akinloye O.A. (2018). First line defence antioxidants-superoxide dismutase (SOD), catalase (CAT) and glutathione peroxidase (GPx): Their fundamental role in the entire antioxidant defence grid. Alex. J. Med..

[81] Martín M.Á., Fernández-Millán E., Ramos S., Bravo L., Goya L. (2014). Cocoa flavonoid epicatechin protects pancreatic beta cell viability and function against oxidative stress. Mol. Nutr. Food Res..

[82] Altun M., Citoglu G.S., Yilmaz B.S., Coban T. (2008). Antioxidant properties of *Viburnum opulus* and *Viburnum lantana* growing in Turkey. Int. J. Food Sci. Nutr..

[83] Zayachkivska O.S., Gzhegotsky M.R., Terletska O.I., Lutsyk D.A., Yaschenko A.M., Dzhura O.R. (2006). Influence of *Viburnum opulus* proanthocyanidins on stress-induced gastrointestinal mucosal damage. J. Physiol. Pharmacol..

[84] Eken A., Yücel O., İpek İ., Ayşe B., Endİrlİk B.Ü. (2017). An investigation on protective effect of *Viburnum opulus* L. fruit extract against ischemia/reperfusion-induced oxidative stress after lung transplantation in rats. Kafkas Univ. Vet. Fak. Derg..

[85] Unusan N. (2020). Proanthocyanidins in grape seeds: An updated review of their health benefits and potential uses in the food industry. J. Funct. Foods.

[86] Naveed M., Hejazi V., Abbas M., Kamboh A.A., Khan G.J., Shumzaid M., Ahmad F., Babazadeh D., FangFang X., Modarresi-Ghazani F. (2018). Chlorogenic acid (CGA): A pharmacological review and call for further research. Biomed. Pharmacother..

[87] Ildiz N., Baldemir A., Altinkaynak C., Özdemir N., Yilmaz V., Ocsoy I. (2017). Self assembled snowball-like hybrid nanostructures comprising *Viburnum opulus* L. extract and metal ions for antimicrobial and catalytic applications. Enzym. Microb. Technol..

[88] Kerry R.G., Patra J.K., Gouda S., Park Y., Shin H.S., Das G. (2018). Benefaction of probiotics for human health: A review. J. Food Drug Anal..

[89] Yetim H., Ekici L., Ozcan C., Ozturk I., Tornuk F., Karaman S. (2017). Effects of some food juices and additives on some physicochemical, textural, color, microbiological and sensory properties of cemen paste. J. Food Process. Preserv..

[90] Şapcı H., Yılmaz F., Vural C., Bahtiyari M.İ., Benli H. (2017). Antimicrobial and antifungal activity of fabrics dyed with *Viburnum opulus* and onion skins. Int. J. Second. Metab..

[91] Yılmaz F., Koçak F., Özgeriş F.B., Şapçı Selamoğlu H., Vural C., Benli H., Bahtiyari M.İ. (2020). Use of *Viburnum opulus* L.(*Caprifoliaceae*) in dyeing and antibacterial finishing of cotton. J. Nat. Fibers.

[92] Altun M.L., Özbek H., Çitoǧlu G.S., Yilmaz B.S., Bayram I., CengIz N. (2010). Hepatoprotective and hypoglycemic activities of *Viburnum opulus* L.. Turk. J. Pharm. Sci..

[93] Peng S.G., Pang Y.L., Zhu Q., Kang J.H., Liu M.X., Wang Z., Huang Y. (2018). Chlorogenic acid functions as a novel agonist of PPARγ2 during the differentiation of mouse 3T3-L1 preadipocytes. Biomed Res. Int..

[94] Kim Y.A., Keogh J.B., Clifton P.M. (2016). Polyphenols and glycémie control. Nutrients.

[95] Clifford M.N., Kerimi A., Williamson G. (2020). Bioavailability and metabolism of chlorogenic acids (acyl-quinic acids) in humans. Compr. Rev. Food Sci. Food Saf..

[96] Ekbatan S.S., Iskandar M.M., Sleno L., Sabally K., Khairallah J., Prakash S., Kubow S. (2018). Absorption and metabolism of phenolics from digests of polyphenol-rich potato extracts using the Caco-2/HepG2 co-culture system. Foods.

[97] Hussain S.A., Sulaiman A.A., Alhaddad H., Alhadidi Q. (2016). Natural polyphenols: Influence on membrane transporters. J. Intercult. Ethnopharmacol..

[98] Tarahovsky Y.S., Kim Y.A., Yagolnik E.A., Muzafarov E.N. (2014). Flavonoid-membrane interactions: Involvement of flavonoid-metal complexes in raft signaling. Biochim. Biophys. Acta-Biomembr..

[99] Corbo F., Brunetti G., Crupi P., Bortolotti S., Storlino G., Piacente L., Carocci A., Catalano A., Milani G., Colaianni G. (2019). Effects of sweet cherry polyphenols on enhanced osteoclastogenesis associated with childhood obesity. Front. Immunol..

[100] Hubert P.A., Lee S.G., Lee S.K., Chun O.K. (2014). Dietary polyphenols, berries, and age-related bone loss: A review based on human, animal, and cell studies. Antioxidants.

[101] Zhang Z., Sun T., Niu J.G., He Z.Q., Liu Y., Wang F. (2015). Amentoflavone protects hippocampal neurons: Anti-inflammatory, antioxidative, and antiapoptotic effects. Neural Regen. Res..

[102] Ovodova R.G., Golovchenko V.V., Popov S.V., Shashkov A.S., Ovodov S.Y. (2000). The isolation, preliminary structural studies, and physiological activity of water-soluble polysaccharides from the squeezed berries of the snowball tree *Viburnum opulus*. Russ. J. Bioorganic Chem..

[103] Torre E. (2017). Molecular signaling mechanisms behind polyphenol-induced bone anabolism. Phytochem. Rev..

[104] Ivanov S.A., Garbuz S.A., Malfanov I.L., Ptitsyn L.R. (2013). Screening of Russian medicinal and edible plant extracts for angiotensin I-converting enzyme (ACE I) inhibitory activity. Russ. J. Bioorganic Chem..

[105] Tuglu D., Yılmaz E., Yuvanc E., Erguder I., Kisa U., Bal F., Batislam E. (2014). *Viburnum opulus*: Could it be a new alternative, such as lemon juice, to pharmacological therapy in hypocitraturic stone patients?. Arch. Ital. Urol. Androl..

[106] Kızılay F., Ülker V., Çelik O., Özdemir T., Çakmak Ö., Can E., Nazlı O. (2019). The evaluation of the effectiveness of gilaburu (*Viburnum opulus* L.) extract in the medical expulsive treatment of distal ureteral stones. Turk. J. Urol..

[107] Dag Z., Akturk G., Filik L. (2014). Acute pancreatitis induced by *Viburnum opulus* juice in a patient with urolithiasis. Asian Pac. J. Trop. Biomed..

[108] Dietz B.M., Hajirahimkhan A., Dunlap T.L., Bolton J.L. (2016). Botanicals and their bioactive phytochemicals for women’s health. Pharmacol. Rev..

[109] Saltan G., Süntar I., Ozbilgin S., Ilhan M., Demirel M.A., Oz B.E., Keleş H., Akkol E.K. (2016). *Viburnum opulus* L.: A remedy for the treatment of endometriosis demonstrated by rat model of surgically-induced endometriosis. J. Ethnopharmacol..

[110] Ulger H., Ertekin T., Karaca O., Canoz O., Nisari M., Unur E., Elmalı F. (2013). Influence of gilaburu (*Viburnum opulus*) juice on 1,2-dimethylhydrazine (DMH)-induced colon cancer. Toxicol. Ind. Health.

[111] Ceylan D., Aksoy A., Ertekin T., Yay A.H., Nisari M., Karatoprak G.Ş., Ülger H. (2018). The effects of gilaburu (*Viburnum opulus*) juice on experimentally induced Ehrlich ascites tumor in mice. J. Cancer Res. Ther..

[112] Zakłos-Szyda M., Pawlik N. (2019). The influence of *Viburnum opulus* polyphenolic compounds on metabolic activity and migration of HeLa and MCF cells. Acta Innov..

[113] Sarıözkan S., Türk G., Eken A., Bayram L.Ç., Baldemir A., Doğan G. (2017). Gilaburu (*Viburnum opulus* L.) fruit extract alleviates testis and sperm damages induced by taxane-based chemotherapeutics. Biomed. Pharmacother..

